# A selective high affinity MYC-binding compound inhibits MYC:MAX interaction and MYC-dependent tumor cell proliferation

**DOI:** 10.1038/s41598-018-28107-4

**Published:** 2018-07-03

**Authors:** Alina Castell, Qinzi Yan, Karin Fawkner, Per Hydbring, Fan Zhang, Vasiliki Verschut, Marcela Franco, Siti Mariam Zakaria, Wesam Bazzar, Jacob Goodwin, Giovanna Zinzalla, Lars-Gunnar Larsson

**Affiliations:** 1 Department of Microbiology, Tumor and Cell Biology, Karolinska Institutet, SE-171 65 Stockholm, Sweden; 2 Present Address: TLV, Box 225 20, 104 22, Stockholm, Sweden; 3 Present Address: Department of Oncology-Pathology, Karolinska Institutet, SE-17176 Stockholm, Sweden

## Abstract

MYC is a key player in tumor development, but unfortunately no specific MYC-targeting drugs are clinically available. MYC is strictly dependent on heterodimerization with MAX for transcription activation. Aiming at targeting this interaction, we identified MYCMI-6 in a cell-based protein interaction screen for small inhibitory molecules. MYCMI-6 exhibits strong selective inhibition of MYC:MAX interaction in cells and *in vitro* at single-digit micromolar concentrations, as validated by split *Gaussia* luciferase, *in situ* proximity ligation, microscale thermophoresis and surface plasmon resonance (SPR) assays. Further, MYCMI-6 blocks MYC-driven transcription and binds selectively to the MYC bHLHZip domain with a K_D_ of 1.6 ± 0.5 μM as demonstrated by SPR. MYCMI-6 inhibits tumor cell growth in a MYC-dependent manner with IC_50_ concentrations as low as 0.5 μM, while sparing normal cells. The response to MYCMI-6 correlates with MYC expression based on data from 60 human tumor cell lines and is abrogated by MYC depletion. Further, it inhibits MYC:MAX interaction, reduces proliferation and induces massive apoptosis in tumor tissue from a MYC-driven xenograft tumor model without severe side effects. Since MYCMI-6 does not affect MYC expression, it is a unique molecular tool to specifically target MYC:MAX pharmacologically and it has good potential for drug development.

## Introduction

The *MYC* family of oncogenes (*MYC*, *MYCN* and *MYCL*, here collectively referred to as “*MYC*”), encodes basic helix-loop-helix leucine zipper (bHLHZip) transcription factors. Through the HLHZip domain, MYC heterodimerizes with the bHLHZip protein MAX, which enables the MYC:MAX complex to bind E-box regulatory DNA elements throughout the genome, thereby controlling transcription of a large group of specific genes^1–6^. The direct target gene products in turn influences global RNA and protein synthesis, thereby coordinating multiple fundamental cellular processes, including cell cycle progression, cell growth, apoptosis, senescence, metabolism and stem cell functions^2,7–11^. Deregulation of expression of MYC family genes/proteins occurs in over half of all human tumors, and is often correlated with aggressive disease, resistance to therapy and poor prognosis^2,4,5,12,13^. MYC is therefore considered as one of the most important drivers of tumor development and has been highlighted as a key therapeutic target for cancer therapy for a number of tumor types. However, so far there are no specific anti-MYC drugs available in the clinic. A number of attempts have been made to target MYC indirectly by interfering with transcription of the *MYC* gene, translation or turnover of the MYC protein or by inhibiting downstream effectors of MYC^14–16^. Due to the diversity of signals regulating the *MYC* genes/proteins and the pleiotropic functions of MYC, tumor cells have multiple ways of escaping these pathways to maintain MYC-family expression and activity. The most reliable strategy is therefore probably to target the MYC proteins directly. Since MYC is strictly dependent on MAX for binding E-boxes, targeting MYC:MAX interaction is a conceivable approach to target MYC. Several examples of successful targeting of protein-protein interactions (PPIs) with small molecules, including Nutlin-3a (targeting p53:MDM2)^17^, BET inhibitors such as JQ1^18^ (bromodomains:histones) and the BH3 mimetic compound Navitoclax/ABT-263 (BCL-2 family interactions)^19^ have been reported recently. These compounds, or improved versions thereof, are now in clinical trials^20,21^, which have encouraged further research on PPIs as drug targets. Several groups have attempted to find compounds targeting the MYC:MAX interaction by screening small-molecule libraries using FRET^22^, fluorescence polarization^23^, or yeast-two-hybrid (Y2H)^24^. As a result, a number of small molecules have been reported to target the MYC:MAX or MYC:MAX:DNA interaction^15,16,22,24–33^. However, none of these compounds have made their way for clinical studies due to a number of limitations including low potency *in vitro* or in cells, poor specificity or inadequate bioavailability *in vivo*^15,16,26,34^. Development of more potent and selective MYC:MAX inhibitors is therefore much warranted. The aim of this work was to identify bioactive molecules that (1) efficiently and selectively inhibit the MYC:MAX interaction *in vitro* and in cells, that (2) bind MYC directly with high affinity, that (3) inhibit MYC-dependent tumor cell growth with high efficacy, that (4) do not affect MYC expression, and that (5) are active *in vivo*. In a cellular MYC:MAX PPI inhibitor screen, we identified the compound MYCMI-6 that fulfills all these criteria.

## Results

### Establishment of a cell-based Bimolecular Fluorescence Complementation (BiFC) assay for screening for small molecules interfering with MYC:MAX protein interactions

**Figure 1:**
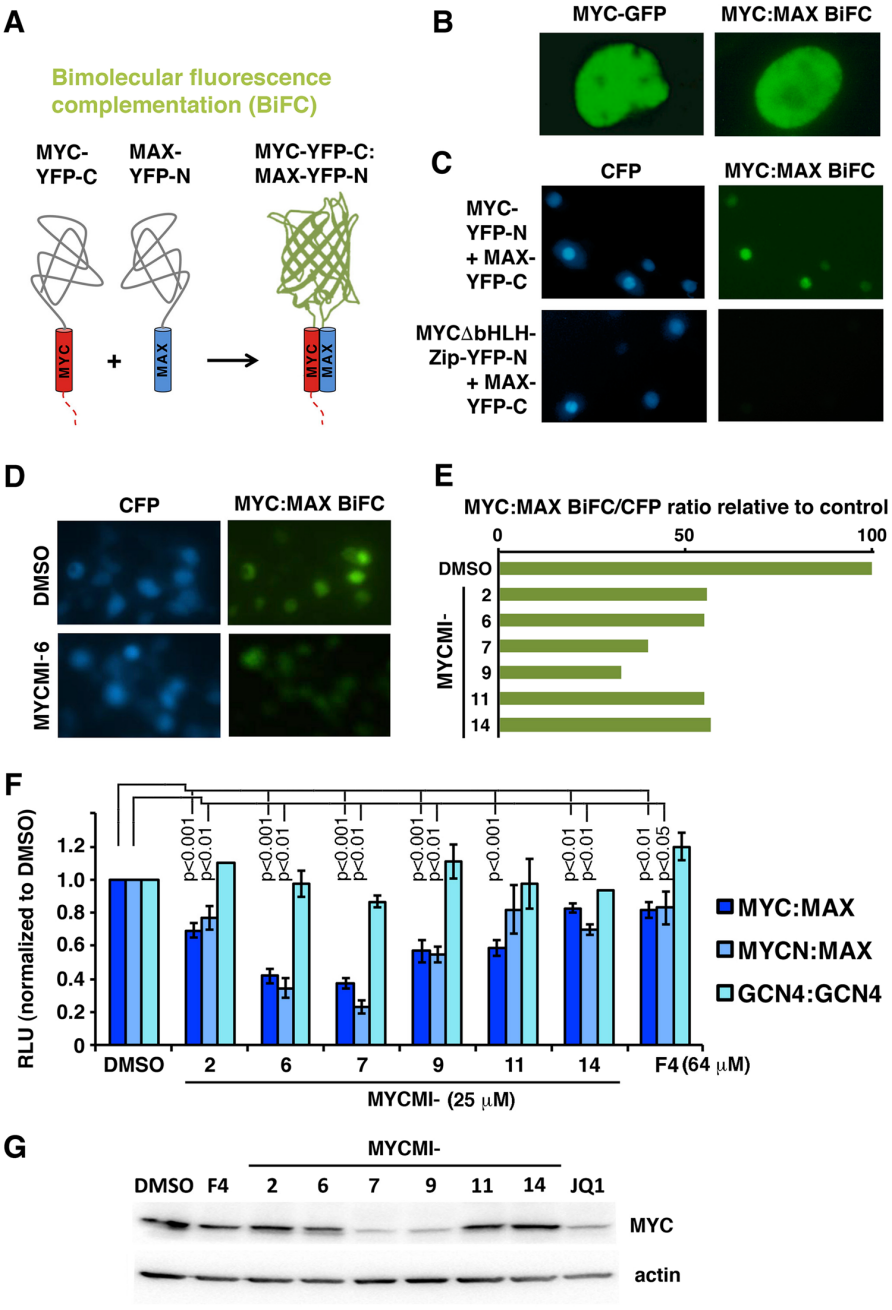
Identification and validation of small molecules targeting the MYC:MAX protein interaction using a cell-based Bimolecular Fluorescence Complementation assay (BiFC). (**A**) Schematic representation of the principle of the BiFC assay. MYC and MAX were fused to two inactive fragments of YFP, generating MYC-YFP-C and MAX-YFP-N, respectively. Upon MYC:MAX heterodimerization, the two YFP fragments refold into a functional YFP protein. (**B**) Nuclear expression of MYC-eGFP (left panel) and MYC-YFP-C and MAX-YFP-N BiFC (right panel). (**C**) HEK293 cells cotransfected with the wt MYC-YFP-C or the MYCΔbHLHZip-YFP-C mutant (lacking the MAX-interacting bHLHZip region of MYC) and MAX-YFP-N BiFC constructs together with CMV-CFP used as internal reference. (**D**) Images of cells treated with compound MYCMI-6 (lower panel) or vehicle (upper panel). CFP channel (left panel) and YFP (BiFC) channel (right panel). (**E**) HEK293 cells cotransfected with MYC-YFP-C and MAX-YFP-N together with CMV-CFP and treated with NCI/DTP Diversity set library (25 μM of each compound) or vehicle (DMSO) for 24 hours in 96-well plates. The ratio of YFP/CFP was calculated relative to DMSO-treated cells. With a cut off of 40% inhibition, six hit compounds (MYCMIs) were identified (**F**) Effect of MYCMIs on MYC:MAX, MYCN:MAX and GCN4:GCN4 interactions using the *Gaussia* luciferase fragment complementation (GLuc) assay. The GLuc fusion protein constructs were transfected into the cells together with the CMV-Luc plasmid and treated with the indicated compounds for 17 hours and analyzed in a dual luciferase assay. The ratio of *Gaussia*/Firefly luciferase luminescence were calculated and normalized to DMSO-treated cells. Data are shown as mean ± standard deviation of 3–7 biological experiments each with 3–6 technical repeats. Significant *p*-values are indicated. (**G**) Western blot analysis of endogenous MYC expression in HeLa cells after 24 hours of treatment with the indicated MYCMIs (10 μM), the experimental MYC:MAX inhibitor 10058-F4^24^ (64 μM) and the BET-inhibitor JQ1^18^ (5 μM). Actin was used as loading control. Full length blots are presented in Suppl. Fig. S9A.

To screen for inhibitors of the MYC:MAX protein interaction, we utilized BiFC^35–37^ to monitor the interaction in living cells. Two constructs encoding complementary BiFC fragments of yellow fluorescent protein (YFP) fused to full length MYC (MYC-YFP-C) and MAX (MAX-YFP-N)^38^, respectively (Figs 1A and S1A), were cotransfected into cells, resulting in a nuclear BiFC signal as a result of MYC:MAX interaction bringing the YFP fragments in close proximity^38^ (Fig. 1B, left panel). To optimize the system for screening purposes, MYC-YFP-C and MAX-YFP-N were transiently coexpressed in HEK293 cells together with a full-length cyan fluorescent protein (CFP) vector as internal control (Fig. 1C, upper panels), after which the ratio of BiFC/CFP fluorescence intensities was calculated. Replacing wt MYC-YFP with a MYC mutant lacking the bHLHZip domain (ΔbHLHZip) required for interaction with MAX, did not produce BiFC signals, thus demonstrating that the MYC:MAX BiFC signal is highly specific (Fig. 1C, lower panels). A strictly standardized mean difference (SSMD) of −5 was calculated from cells expressing CFP together with MYC:MAX BiFC and MYCΔbHLHZip:MAX BiFC, respectively, suggesting a strong threshold for hit selection. We concluded that this system was suitable for screening small molecule libraries to identify inhibitors of MYC:MAX interaction in cells.

### Identification and validation of small molecules selectively targeting the MYC:MAX interaction in cells

We next utilized a diversity set library from the NCI/DTP Open Chemical Repository (http://dtp.cancer.gov) consisting of 1990 compounds to screen for small molecules reducing the MYC:MAX BiFC signal relative to the CFP signal as an indication of potential MYC:MAX inhibition in cells. Compounds (25 μM) decreasing the BiFC/CFP fluorescence ratio by more than the arbitrary threshold of 40% relative to the vehicle (DMSO) control after 24 hours treatment were considered as positive hits. Six compounds fulfilling this criterion (Fig. 1E), while not affecting the interaction between the bZip transcription factors FOS and JUN (Suppl. Fig. S2A), were chosen for further studies and were dubbed MYCMIs (**MYC**:**M**AX **I**nhibitors).

For validation, we established another cellular protein interaction assay based on the use of complementary fragments of the *Gaussia* luciferase (GLuc)^39^ fused to full length MYC (MYC-GLuc-C) and MAX (MAX-GLuc-N), respectively (Suppl. Fig. S1B). Cotransfection of HEK293 cells with these constructs together with Firefly luciferase in a dual luciferase assay resulted in a high relative GLuc activity, while a mutant MYC-GLuc-C protein lacking the Zip interaction domain (MYCΔZip) gave only background activity, thus demonstrating the specificity of the system (Yan *et al*., manuscript in prep.). Treatment of cotransfected cells with the hit molecules (25 μM) showed that all six compounds significantly inhibited MYC:MAX GLuc activities (Fig. 1F), most of them more efficiently than the previously reported MYC:MAX inhibitor 10058-F4^24^, which was used at a concentration of 64 μM. The most potent among the molecules were MYCMI-6 and MYCMI-7. All compounds inhibited MYCN:MAX GLuc activity to a similar extent as MYC:MAX, but did not reduce GLuc activity of the homodimeric bZIP transcription factor GCN4, thus showing selectivity for MYC family protein:MAX interactions (Fig. 1F).

**Figure 2:**
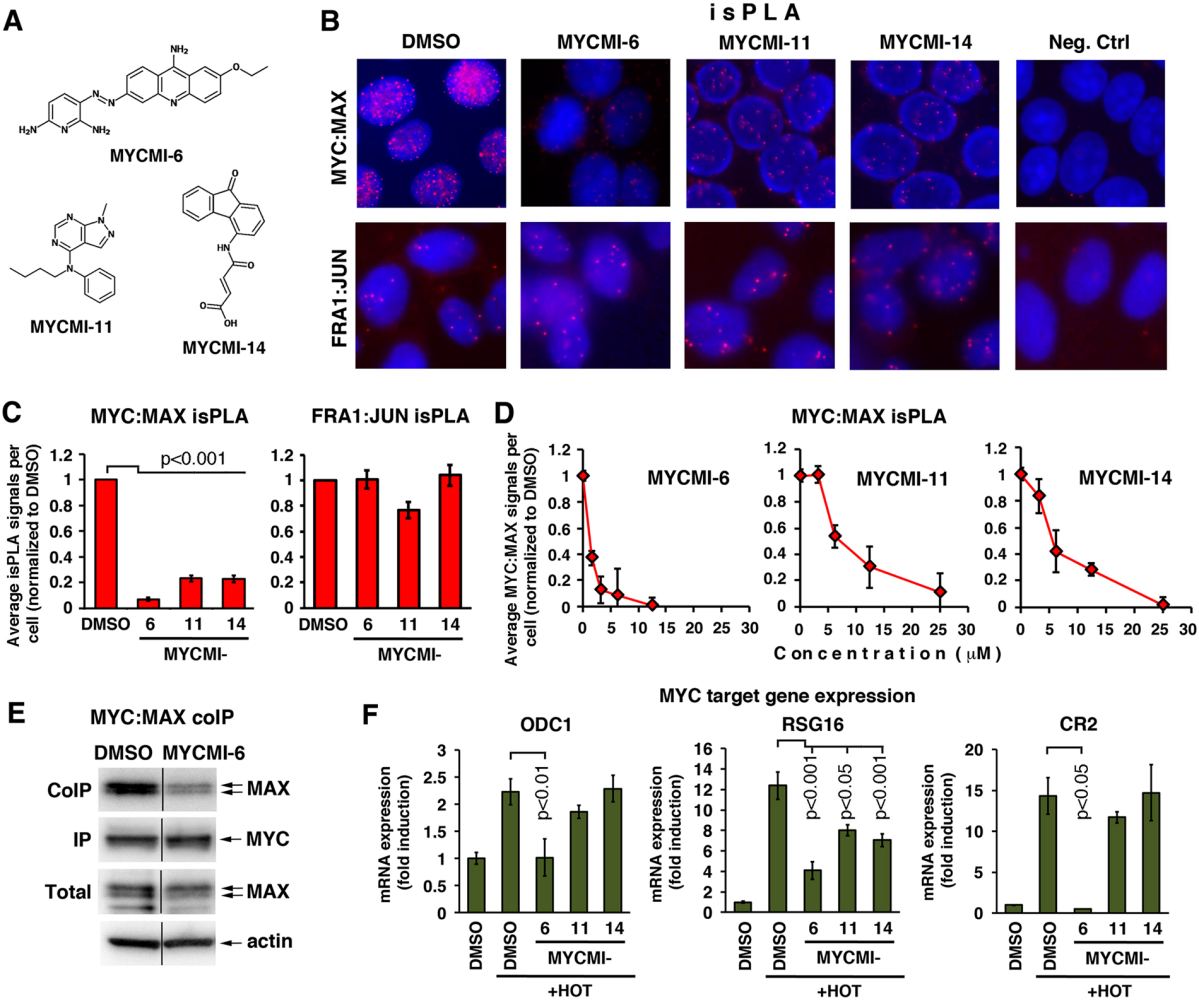
MYCMIs inhibit endogenous MYC:MAX interaction in breast cancer cells and repress MYC-induced target gene expression. (**A**) Chemical structures of MYCMI-6, MYCMI-11 and MYCMI-14. (**B**–**D**) *in situ* Proximity Ligation Assay (isPLA). (**B**) Endogenous MYC:MAX (upper panel) and FRA1:JUN (lower panel) interactions visualized by isPLA as fluorescent red dots in cell nuclei (blue) after treatment with indicated compounds (10 μM) or DMSO for 16 hours. isPLA was performed using pairs of MYC and MAX and of FRA1 and JUN antibodies, respectively. As negative control, one primary antibody was used together with the pair of secondary antibodies. The isPLA results are based on three biological experiments for MYC:MAX and two for FRA1:JUN. One representative experiment for each is shown. (**C**) Quantification of MYC:MAX (left panel) and FRA1:JUN (right panel) isPLA, representing an average number of nuclear dots per cell from three microscopic fields normalized to corresponding values for DMSO-treated cells. *p*-values are indicated. (**D**) Titration of indicated compounds in MCF7 cells for 24 hours prior to MYC:MAX isPLA assay. Quantification was performed as in (**C**). (**E**) Coimmunoprecipitation of endogenous MAX with MYC from MDA-MB231 cells treated with 5 μM MYCMI-6 or DMSO for 3.5 hours. 1^st^–4^th^ lanes from top; coimmunoprecipitated MAX, immunoprecipitated MYC, total levels of MAX and ACTIN, respectively, as determined by western blot analysis. Note that the gels have been cropped. The uncropped, full length versions are presented in Suppl. Fig. S9B. (**F**) Inhibition of MYC transactivation of target genes ODC1, RSG16, and CR2 as determined by RT-qPCR analysis, based on three biological experiments with three technical repeats each. U2OS-MYC-ER cells were treated with or without 100 nM 4-hydroxy-tamoxifen (HOT) for 4 hours, after which DMSO or indicated compounds (10 μM) were added for 24 hours before total RNA was extraction. Fold changes in mRNA expression are presented relative to DMSO in non-HOT-treated cells after normalization to GAPDH, used as reference gene. Significant *p*-values are indicated.

Next, the effect of the compounds on endogenous MYC protein levels was examined. Two of the compounds, MYCMI-7 and MYCMI-9, reduced MYC protein expression as determined by western blot analysis (Fig. 1G). We focus this paper on three compounds, MYCMI-6 (NSC354961), MYCMI-11 (NSC11656) and MYCMI-14 (NSC49689) (Fig. 2A) that did not affect MYC protein levels to any greater extent, with the aim to identify compounds specifically targeting MYC:MAX interaction without interfering with other MYC activities.

### MYCMI-6 selectively inhibits endogenous MYC:MAX interaction and MYC-induced gene expression

To validate the inhibitory effects of the selected MYCMIs on endogenous MYC:MAX interaction in cells, *in situ* proximity ligation assay (isPLA)^40^ was performed using MYC and MAX antibodies. The interactions were visualized as fluorescent dots mainly localized in the cell nucleus by fluorescence microscopy (Fig. 2B) as previously reported^40^. Treatment of breast cancer cells with the MYCMI-6, MYCMI-11 and MYCMI-14 for 24 hours significantly decreased MYC:MAX isPLA signals to 7%, 23% and 23% of DMSO-treated controls, respectively (Fig. 2B and C). Titration showed an IC_50_ for inhibition of MYC:MAX of less than 1.5 μM for MYCMI-6 and of approximately 6 μM for MYCMI-11 and MYCMI-14 by isPLA (Fig. 2D). Further, coimmunoprecipitation of endogenous MYC:MAX proteins showed that MYCMI-6 reduced the MYC:MAX protein interaction already at 3 hours post treatment (Fig. 2E). In contrast, the three compounds did not significantly affect the interaction between the bZip transcription factors FRA1 and JUN (Fig. 2B and C) or the interaction between MAX and the bHLHZip protein MXD1(MAD1) – an intracellular competitor of MYC for MAX^41,42^ (Suppl. Fig. S2B), thus further supporting MYC:MAX selectivity of the compounds.

To investigate the effect of the compounds on MYC-driven transcription, we utilized U2OS cells expressing a MYC-estrogen receptor (MYC-ER) fusion protein, the activity of which is regulated by the ligand 4-hydroxytamoxifen (HOT)^43^. Treatment with MYCMI-6 significantly reduced HOT-induced expression of three previously described direct MYC target genes, ODC1, RSG16 and CR2^10,11^, while MYCMI-11 and -14 in general reduced expression of the genes to a lesser extent, being significant only for RSG16 (Fig. 2F). Taken together, these results highlight MYCMI-6 as the most potent and selective inhibitor of endogenous MYC:MAX protein interactions and of MYC-driven transcription among the three MYCMIs.

### MYCMI-6 is a potent and selective inhibitor of the MYC:MAX bHLHZip interaction *in vitro*

**Figure 3:**
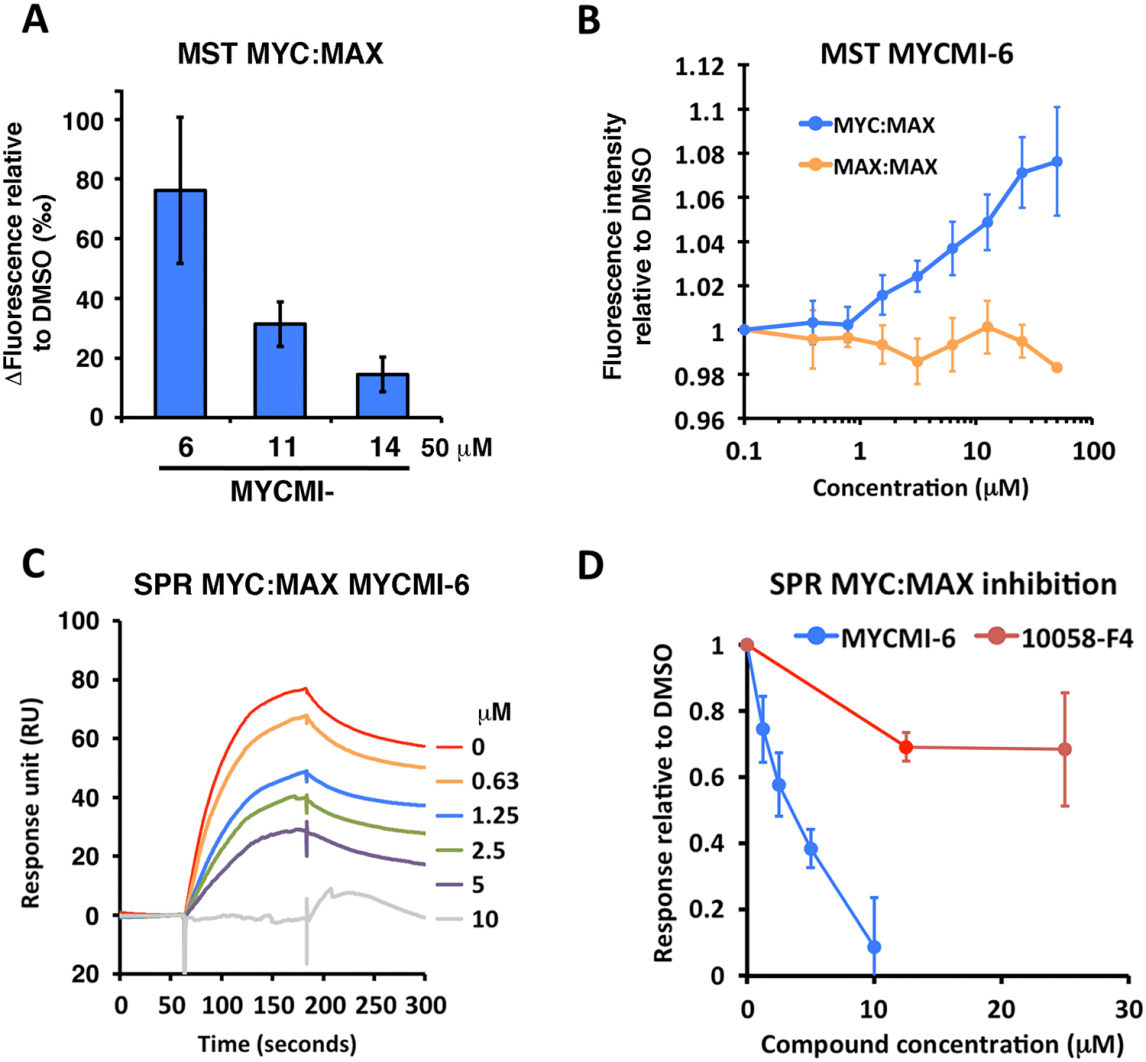
MYCMI-6 inhibits the MYC:MAX bHLHZip protein interaction *in vitro*. (**A**) Microscale thermophoresis (MST) of fluorescently labeled MAX in a MYC:MAX heterodimer formation assay based on recombinant proteins. 1 µM MYCbHLHZip was pre-incubated with 50 µM of respective MYCMI before mixing with 0.5–1 µM labeled MAXbHLHZip. MST was induced and the relative changes in fluorescence (thermophoresis of labeled MAX) to DMSO were analyzed and compared. (**B**) MST of labeled MAXbHLHZip after titration of MYCMI-6 pre-incubated with 1 µM MYCbHLHZip, or with 1 µM MAXbHLHZip. Fluorescence intensity of labeled MAXbHLHZip relative DMSO was plotted against MYCMI-6 concentration. 3–4 experiments were carried out each with technical duplicates. (**C**) Surface plasmon resonance (SPR) of MYC:MAX heterodimer formation assay. MAXbHLHZip was immobilized by an amino coupling procedure to a CM5 sensor chip. MYCbHLHZip pre-incubated with or without compound (as indicated) was injected over MAX for 180 seconds, allowed to dissociate for 240 seconds and regenerated. Reference surface (without MAXbHLHZip) subtracted sensorgrams are shown from one representative experiment. (**D**) SPR of MYC:MAX. Binding levels of MYCbHLHZip to MAXbHLHZip were analyzed and plotted against concentration of MYCMI-6, or 10058-F4, respectively. Three experiments were carried out, respectively.

We next investigated the capacity of the small molecule inhibitors to directly target the MYC:MAX interaction *in vitro*. Two biophysical interaction assays, microscale thermophoresis (MST)^44^ and surface plasmon resonance (SPR)^45^, were developed using recombinant MYC and MAX proteins (Suppl. Fig. S1C) (manuscript in prep.). MST is based on the shift of fluorescent molecules moving in a temperature gradient (thermophoresis), which can be perturbed by interactions. In the MST MYC:MAX interaction assay, fluorescently labeled MAXbHLHZip was combined with MYCbHLHZip pre-mixed with compound. All three MYCMIs, and in particular MYCMI-6, shifted MAX thermophoresis relative to DMSO, indicating that the compounds affected the MYC:MAX conformation (Fig. 3A). Titration of MYCMI-6 in mixture with MYC and labeled MAX resulted in a thermophoresis shift with a K_d_ of 4.3 +/− 2.9 μM, while having minor effects on labeled MAX when pre-mixed with unlabeled MAX (Fig. 3B), suggesting that MYCMI-6 discriminates well between MYC:MAX and MAX:MAX interactions.

The SPR method is a highly sensitive assay to determine the affinity between protein and ligand and to measure the kinetics of the interaction^45^. In the SPR MYC:MAX interaction assay, MAXbHLHZip was covalently attached onto a sensor chip and MYCbHLHZip pre-mixed with compound was injected over the surface and thereafter allowed to dissociate. MYCMI-6 inhibited the MYC:MAX heterodimer formation with an IC_50_ of 3.8 + /− 1.2 μM (Fig. 3C and D), whereas the experimental MYC inhibitor 10058-F4 was less efficient (Figs 3D and S3A). The previously identified MYC:MAX interaction inhibitor KJ-Pyr-9^27^ had no effect on MYC:MAX formation up to 10 μM (Supp. Fig. S3B).

### MYCMI-6 binds directly and selectively to the MYC bHLHZip domain with high affinity

**Figure 4:**
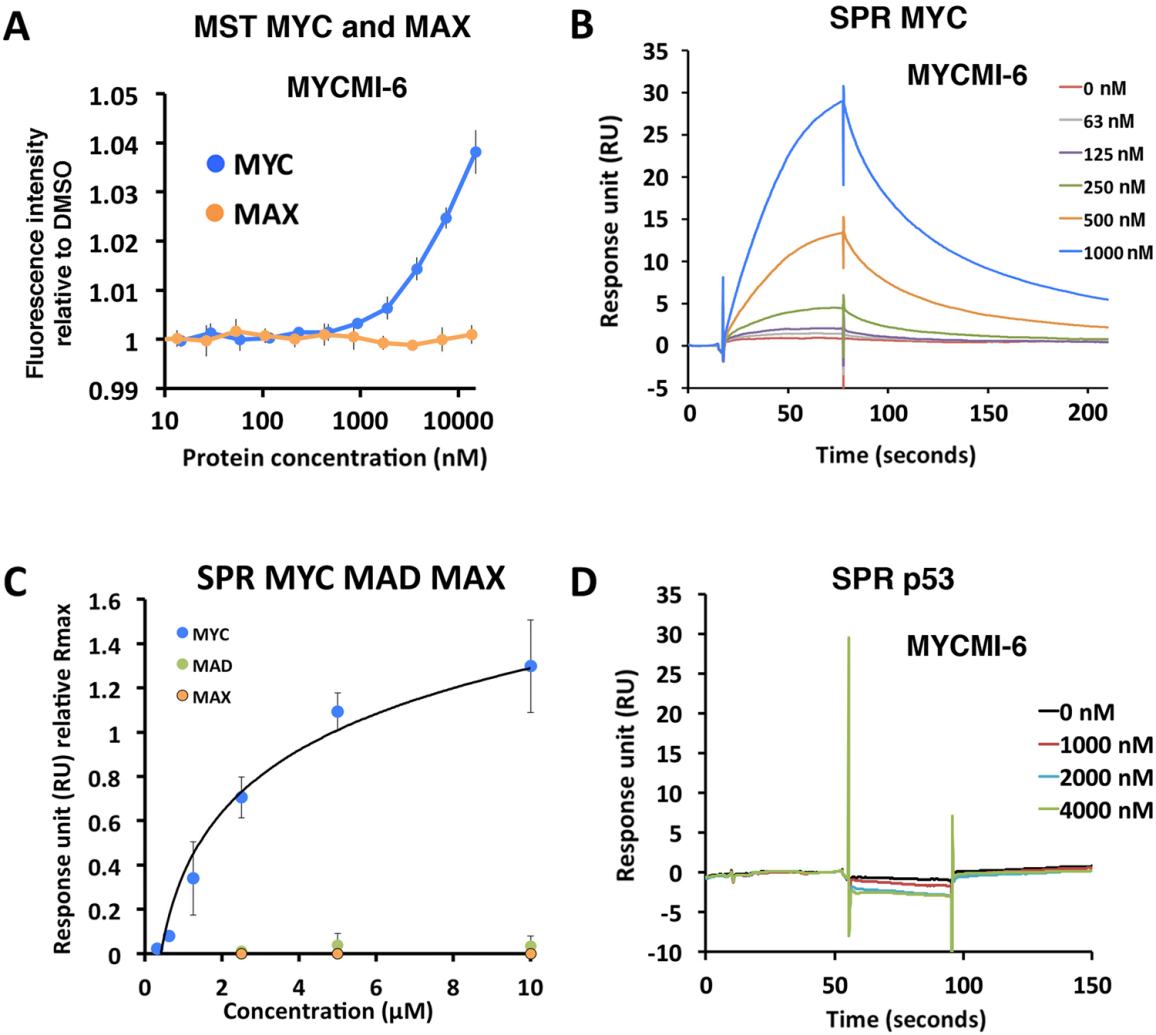
MYCMI-6 binds selectively and with high affinity to the MYC bHLHZip domain. (**A**) MST assay measuring the effect of MYCMI-6 on MYC and MAX, respectively. Recombinant MYC bHLHZip and MAX bHLHZip proteins were titrated, respectively, in a fixed concentration (3 µM) of MYCMI-6. Changes in fluorescence were measured and normalized to control (buffer). Data are shown as mean ± standard deviation of 6–8 biological repeats. (**B**) SPR assay to determine the affinity of MYCMI-6 to MYC. MYC bHLHZip protein was immobilized by amino coupling on a CM5 sensor chip. MYCMI-6 was injected at various concentrations in a kinetic experiment. The reference surface was subtracted from the analyte surface to generate a sensorgram. Association and dissociation rates (k_a_ = 9294 M−1 s−1, k_d_ = 0.02293 s−1) were determined using the Langmuir 1:1 model in the Biacore Evaluation program fitting curves with a constant Rmax of 43 RU (theoretical Rmax), thereby suggesting a K_D_ of 2.5 µM with a Chi^2^ value of 0.073. The sensorgram displays one representative experiment. Four kinetic experiments were carried out on two different sensor chips and an average K_D_ of 1.6 ± 0.5 µM was calculated. (**C**) Four MYC equilibrium binding experiments with MYCMI-6 summarized in an equilibrium binding plot, carried out on two different sensor chips. Binding affinities were estimated from the plot as 50% of Rmax suggesting a K_D_ of approximately 1.5–2 μM with an experimental Rmax of 25–30 RU (theoretical Rmax = 23 RU). SPR experiments of MYCMI-6 binding to immobilized MXD1 (MAD1) and MAX protein, respectively were included in the graph. Sensorgrams are shown in Suppl. Fig. S5A,C and D. (**D**) Reference subtracted sensorgram from the p53 protein SPR assay. p53 core protein was immobilized to 3000 RU and MYCMI-6 was injected at various concentrations (theoretical Rmax of 56 RU).

To determine whether MYCMI-6 binds directly to MYC (or MAX), MST and SPR were applied. The bHLHZip domains of MYC or MAX, respectively (Suppl. Fig. S1C), were titrated and mixed with 3 μM of MYCMI-6. There were clear changes in thermophoresis at 1–15 μM of MYC while no obvious changes were observed for MAX, indicating binding of MYCMI-6 to MYC but not to MAX at these concentrations (Fig. 4A). Further, MYCMI-6 did not bind the control proteins BCL-X_L_ (Suppl. Fig. S4A) and bovine serum albumin (BSA) (Suppl. Fig. S4B). Similar results were obtained in a reverse MST experiment, keeping labeled MYC at a fixed concentration while titrating MYCMI-6, showing relative changes in thermophoresis at concentrations above 100 nM, supporting that MYCMI-6 binds MYC with affinity in the low micromolar range (Suppl. Fig. S4C).

Direct binding of MYCMI-6 to MYC was also analyzed by SPR, where MYC bHLHZip was immobilized on the chip and different concentrations of MYCMI-6 were injected in kinetic experiments. Association and dissociation rates of the compound were obtained from a 1:1 Langmuir model after subtraction of reference cell values, suggesting a K_D_ value of 1.6 ± 0.5 μM (Fig. 4B). From equilibrium binding experiments (Suppl. Fig. S5A), a K_D_ value of approximately 1.5–2 μM was determined by an equilibrium binding plot (Fig. 4C). The previously identified MYC:MAX interaction inhibitors 10074-G5 and #474 (an analogue of 10058-F4)^32^ used as reference compounds were found to bind to MYC with K_D_s of 28 and 15 μM, respectively (data not shown). We could, however, not detect any binding of KJ-Pyr-9 up to 8 μM in this assay (Suppl. Fig. S5B). Further, MYCMI-6 binding to MAX, MXD1 (MAD1) (Figs 4C and S5C and D), p53 (Fig. 4D), BSA (Suppl. Fig. S5E) and YFP (data not shown) were negligible. Binding to BCL-X_L_ was only 10% of the theoretical maximum at 2 μM MYCMI-6 (Suppl. Fig. S5F). In summary, the MST and SPR experiments suggest that MYCMI-6 binds the bHLHZip domain of MYC directly, selectively and with affinity in the low micromolar range.

### MYCMI-6 selectively suppresses MYC-driven tumor cell growth with high efficacy

**Figure 5:**
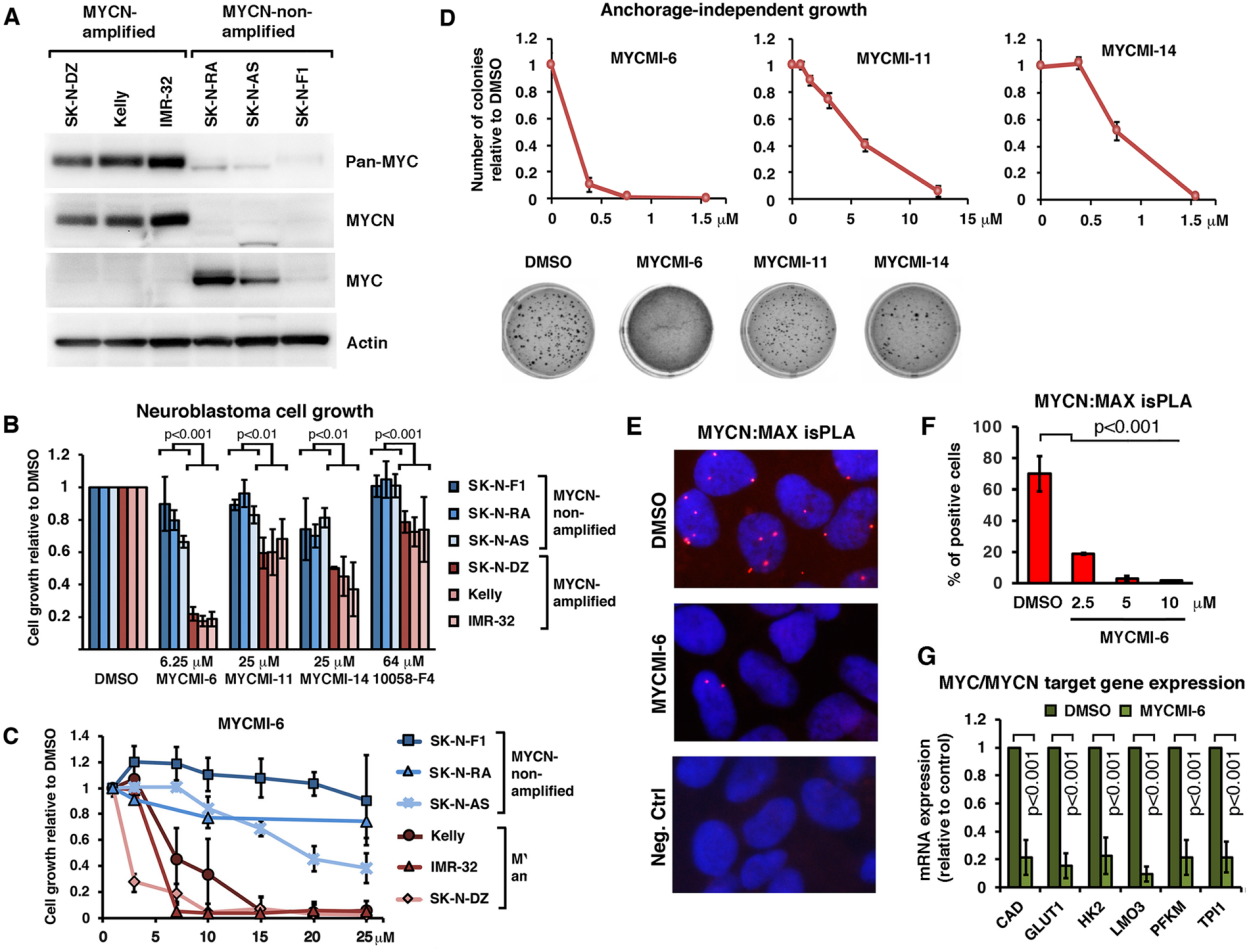
MYCMIs preferentially inhibit growth of *MYCN*-amplified compared to non-amplified neuroblastoma cells correlating with MYC family protein expression. (**A**) Western blot analysis of MYCN, MYC and pan-MYC protein expression in *MYCN*-amplified neuroblastoma cells (IMR-32, Kelly and SK-N-DZ) and *MYCN*-non-amplified neuroblastoma cells (SK-N-F1, SK-N-AS and SK-N-RA). Pan-MYC antibodies recognizing all MYC family proteins or antibodies specific for MYCN or MYC (see Supplementary Information), respectively, were used as indicated. Full length versions of the gels are presented in Suppl. Fig. S10. (**B**) Indicated neuroblastoma cell lines were treated with MYCMI-6 (6.25 μM), MYCMI-11 and MYCMI-14 (25 μM), reference compound 10058-F4 (64 μM) or DMSO control for 48 hours after which cell growth/viability was measured by the resazurin assay. Data are shown as mean ± standard deviation of 2–5 biological repeats. *p*-values are indicated. (**B**) Titration of MYCMI-6 onto three *MYCN*-non-amplified and three *MYCN*-amplified cell lines as indicated for 48 hours followed by the resazurin assay. (**C** and **D**) Anchorage-independent cell growth of *MYCN*-amplified neuroblastoma cells. (**C**) Anchorage-independent growth of neuroblastoma SK-N-DZ cells in 0.35% agarose in 24 well plates in the presence of DMSO or compounds at indicated concentrations. After 16 days, colonies were stained with MTT and the numbers of colonies were counted. Images of colonies in wells treated with 0.75 μM of compound, analyzed after two weeks, are displayed (lower panel). (**E** and **F**), visualization of endogenous MYCN:MAX interaction by isPLA in *MYCN*-amplified SK-N-BE(2) cells, performed as described in the legend to Fig. 2 using MYCN and MAX antibodies. (**E**) Images of the isPLA assay. The cells were treated with MYCMI-6 (2.5 µM) or DMSO for 16 hours. As negative control, one primary antibody was used together with the pair of secondary antibodies. (**F**) Quantification of MYCN:MAX isPLA, representing the average percentage of cells displaying MYCN:MAX nuclear dots from three microscopic fields after treatment with MYCMI-6 at indicated concentrations for 16 hours normalized to corresponding values for DMSO-treated cells. *p*-values are indicated. (**G**) RT-qPCR analysis of mRNA expression of selected MYC/MYCN target genes^47^, in *MYCN*-amplified Kelly cells after treatment with MYCMI-6 (2.5 μM) or DMSO for 24 hours. The analysis is based on three biological experiments with three technical repeats each. Fold changes in mRNA expression in response to MYCMI-6 are presented relative to DMSO after normalization to total RNA per cell. *p*-values are indicated.

We next addressed if treatment with MYCMI-6, -11 and -14 inhibits tumor cell growth and if this correlates with the MYC status of the cells. For this purpose, we first utilized a panel of neuroblastoma cell lines with or without *MYCN*-amplification. Since a subset of *MYCN*-non-amplified neuroblastomas have been shown to exhibit deregulated expression of (c)-MYC^46^, we investigated the expression of MYCN and MYC as well as total level of MYC-family proteins in the six cell lines using antibodies specific for MYCN, MYC or pan-MYC. While the three cell lines with *MYCN*-amplification all expressed high levels of MYCN protein, two out of three of the *MYCN*-non-amplified cell lines expressed MYC, although the total level of MYC-family proteins were much lower in these cells than in the *MYCN*-amplified cell lines (Fig. 5A). The cell lines were exposed to MYCMI-6 (6.25 μM), MYCMI-11 or MYCMI-14 (25 μM) or the reference 10058-F4 (64 μM) for 48 hours. All four compounds reduced growth of the *MYCN*-amplified cell lines significantly stronger than the *MYCN*-non-amplified cell lines (Fig. 5A). Titration experiments confirmed that MYCMI-6 discriminates between *MYCN*-amplified and *MYCN*-non-amplified neuroblastoma cell lines cells, with average growth inhibition of 50% (GI_50_) values of 2.5–6 μM for *MYCN*-amplified cell lines and around 20 μM or higher for *MYCN*-non-amplified cell lines (Fig. 5B). Notably, in the SK-N-F1 cell line, which essentially did not respond at all to MYCMI-6, MYCN or MYC were hardly detectable. These results demonstrate that the response to MYCMI-6 and the other MYCMIs in general correlated well with the total level of MYC-family proteins. Further, the MYCMI-6, MYCMI-11 and MYCMI-14 efficiently inhibited anchorage-independent growth of *MYCN*-amplified neuroblastoma cells with GI_50_ values of <0.4, 5 and 0.75 μM, respectively (Fig. 5C and D). These results demonstrate that MYCMI-6 is clearly the more potent and selective of the MYCMIs, and we therefore focused the remainder of this work on MYCMI-6.

To verify that MYCMI-6 inhibited MYCN:MAX interaction and the transcriptional output of MYCN in *MYCN*-amplified neuroblastoma cells, we performed MYCN:MAX isPLA and measured the expression of a panel of verified MYC family target genes in neuroblastoma^47^ in response to MYCMI-6. Figure 5E–G shows that MYCMI-6 significantly inhibited MYCN:MAX interaction and the expression of MYC/MYCN target genes already at a concentration of 2.5 μM, while maintaining the expression of MYCN and MAX (Suppl. Fig. S6). This is consistent with the results from MYCN:MAX GLuc interaction assays after MYCMI-6 treatment (Fig. 1F).

**Figure 6:**
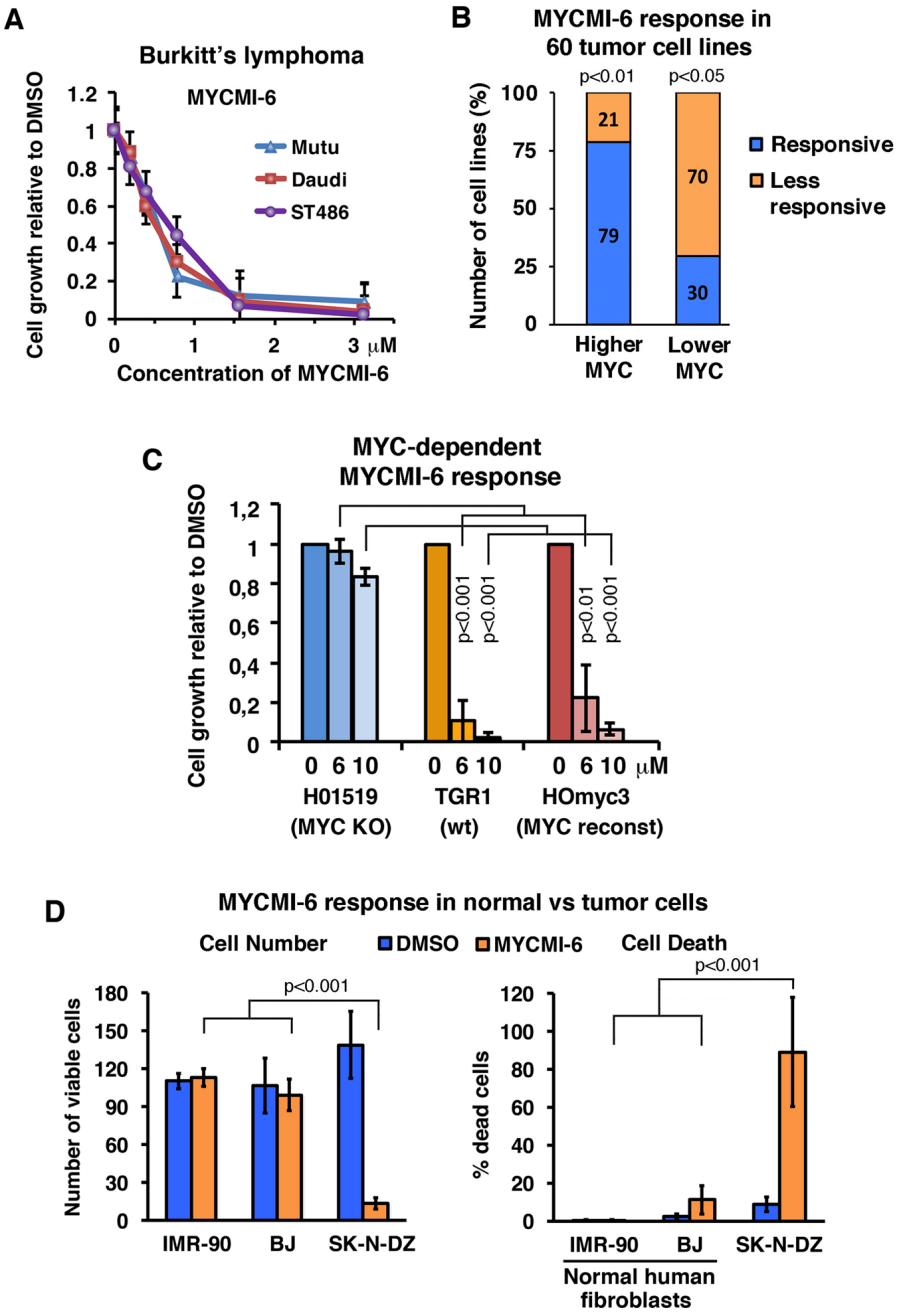
MYCMI-6 inhibits tumor cell growth and viability in a MYC-dependent manner but is not cytotoxic to primary normal human cells. (**A**) MYCMI-6 titration on Burkitt’s lymphoma (BL) cell lines Mutu, Daudi and ST486. Data are shown as mean ± standard deviation of 2 biological experiments, each with 3 technical repeats. (**B**) Correlation MYCMI-6 response (GI_50_) with MYC mRNA levels of the NCI-60 human tumor cell lines extracted from CellMiner™ and complemented with MYC protein levels from the Novartis proteome scout project or from the literature (see Supplementary Table S1). “Responsive” and “less responsive”; cell lines with positive and negative log 10 GI_50_ values, respectively. “Higher MYC” and “lower MYC”; cell lines with higher and lower MYC expression levels (MYC mRNA/protein) than average, respectively. *p*-values are indicated. (**C**) Growth of TGR-1 (wt), HO15.19 (MYC knockout) and HOmyc3 (MYC reconstituted HO15.19) Rat1 fibroblasts, as measured by the WST-1 assay after treatment with MYCMI-6. Data are shown as mean ± standard deviation of 3–5 biological experiments, each with 3 technical repeats. *p*-values are indicated. (**D**) Normal IMR-90 and BJ human fibroblasts and the *MYCN*-amplified neuroblastoma cell line SK-N-DZ were treated with 12.5 μM MYCMI-6 or control (DMSO) for 24 hours. The number of viable and percentage of dead cells were quantified by addition of CellTracker Green (stains all cells) and DAPI (stains dead cells) to cells and analyzed in GFP and CFP channels using a fluorescence microscope. *p*-values are indicated.

Moreover, MYCMI-6 significantly inhibited growth of Burkitt’s lymphoma (BL) cells - another classical example of a MYC-driven tumor, having translocations of *MYC* to one of the immunoglobulin loci - in a dose-dependent manner with an average GI_50_ of 0.5 μM (Fig. 6A).

To further investigate whether the levels of MYC expression in tumor cells correlate with the growth inhibitory response, we utilized GI_50_ data from the NCI-60 diverse human tumor cell line panel available for MYCMI-6 by the Developmental Therapeutics Program of the U.S. National Cancer Institute (DTP-NCI), extracted from the NIH-supported CellMiner™ version 2.1 (https://discover.nci.nih.gov/cellminer). Since deregulated MYC expression is frequently manifested at the protein level, for instance through protein stabilization or deregulated mRNA translation^14,48,49^, we complemented the analysis with MYC protein data obtained from Novartis proteome scout SymAtlas Project (https://proteomescout.wustl.edu/proteins/52581/expression) or elsewhere in the literature (for details, see Supplementary Table S1), and separated cell lines with high or low MYC mRNA and/or high protein level into the categories “higher MYC” and “lower MYC” cell lines, respectively, as indicated in Supplementary Table S1 and Suppl. Fig. S7A and B. There was a significant correlation between the response to MYCMI-6 and the MYC mRNA/protein levels among the 60 tumor cell lines, whereas an analysis based on MYC mRNA alone did not reach significance (Suppl. Fig. S7A and B). Categorizing the cell lines as “responsive” or “less responsive” to MYCMI-6 based on average logarithmic GI_50_ values, we found that 79% of the cell lines with higher MYC expression showed a good growth inhibitory response to MYCMI-6, while 70% of the cell lines with lower MYC expression were less responsive (Fig. 6B). Statistical analysis showed that the probability of a “higher MYC” cell line being responsive was significantly higher than not being responsive, while the probability of a “lower MYC” cell line to be less responsive was significantly higher than being responsive (Fig. 6B), thus demonstrating a strong correlation between MYC expression and growth inhibitory response to MYCMI-6 in human cancer cell lines.

### MYCMI-6 treatment reduces cell growth and survival in a MYC-dependent manner but is not cytotoxic to normal human cells

To further explore the MYC-dependence of MYCMI-6 cell growth inhibitory responses, we utilized immortal H015.19 MYC null rat fibroblasts derived from Tgr1 (parental cells) and H0Myc3 cells, which has been reconstituted with MYC^50^. MYCMI-6 strongly inhibited growth of wt and reconstituted cells, but did not significantly affect growth of the MYC null cells, thus showing a clear difference in response between MYC expressing and MYC-deficient cells (Fig. 6C), thus further supporting the conclusion that the cellular response after MYCMI-6 treatment is MYC-dependent.

We next addressed the question whether cellular responses to MYCMI-6 treatment differentiate between MYC-dependent tumor cells and normal primary human cells. As shown in Fig. 6D, MYCMI-6 (12.5 μM) dramatically increased cell death and reduced cell number in *MYCN*-amplified SK-N-DZ neuroblastoma cells after 24 hours treatment but had only marginal effects on the number of viable cells and the percentage of dead cells in normal lung (IMR-90) and foreskin (BJ) human fibroblasts. We concluded that MYCMI-6 was well tolerated by the normal human cells at a concentration that was highly toxic to MYC-dependent tumors cells, showing good discrimination between cancer cells and normal cells in response to MYCMI-6 resulting in a good therapeutic window for this compound.

### MYCMI-6 induces massive apoptosis and reduces tumor cell proliferation, tumor microvasculature density and MYC:MAX interaction in a MYC-dependent xenograft tumor model *in vivo*

**Figure 7:**
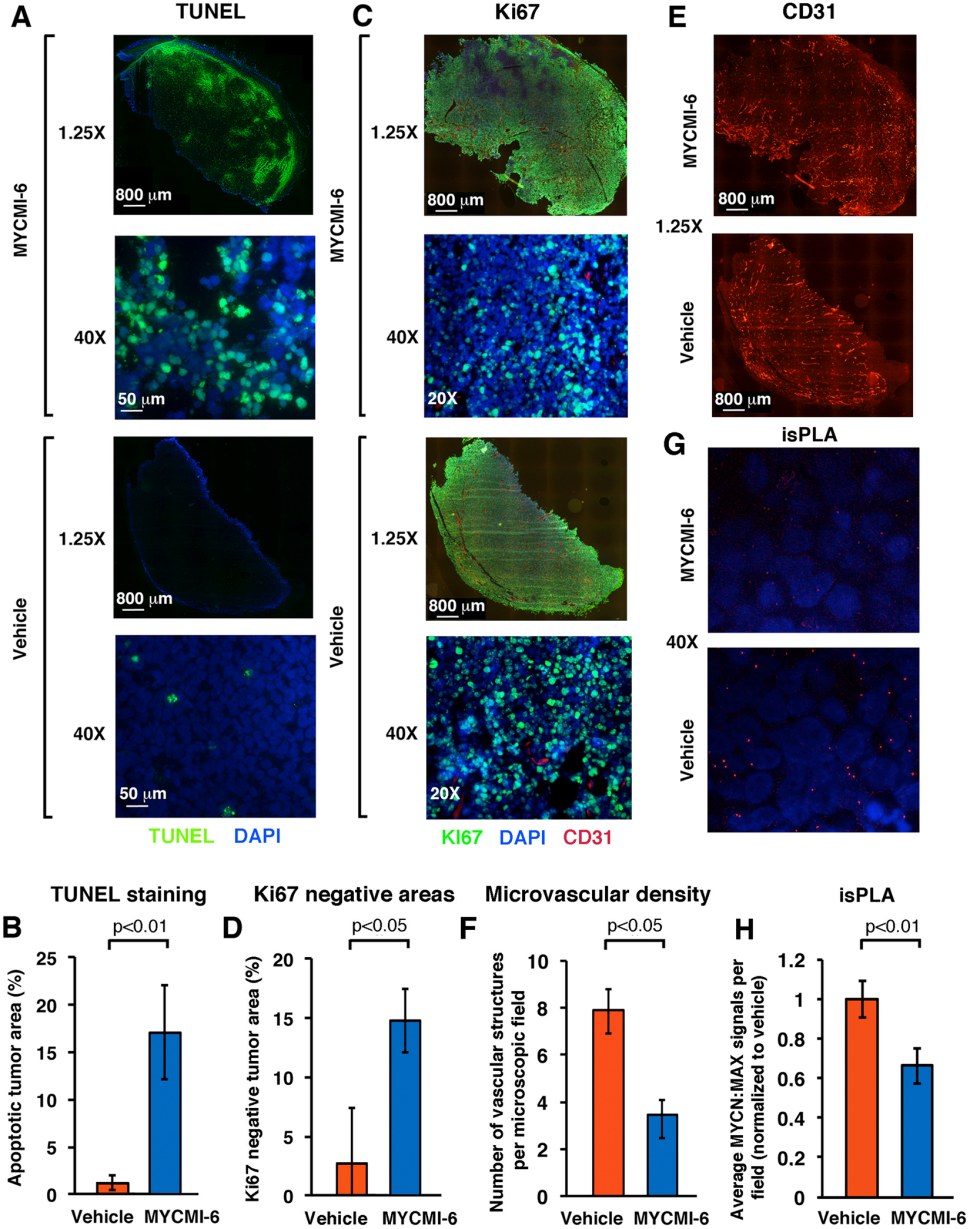
MYCMI-6 inhibits MYC:MAX interaction, induces apoptosis and reduces tumor cell proliferation and microvascularity in a *MYCN*-amplified neuroblastoma mouse tumor model *in vivo*. SK-N-DZ *MYCN*-amplified neuroblastoma xenograft tumors reaching a volume of 100–200 mm^3^ were treated with MYCMI-6 (20 mg/kg body weight) or vehicle injected i.p. daily for 1–2 weeks. (**A**) Apoptosis was determined by TUNEL staining (green) of tumor tissues from mice treated with MYCMI-6 (upper two panels) or vehicle (lower two panels), counterstained with DAPI (blue). Representative images are shown at 1.25X (panel 1 and 3 from top, bar = 800 μM) or 40X (panel 2 and 4, bar = 50 μM) magnification. (**B**) Quantification of TUNEL staining normalized to whole tumor areas as determined by DAPI from three MYCMI-6- and three vehicle-treated mice. (**C**) Cell proliferation and microvascular density determined by Ki67 (green) and CD31 (red) staining, respectively, of tumor tissues from mice treated with MYCMI-6 (upper two panels) or vehicle (lower two panels), respectively, and counterstained with DAPI (blue). Representative images taken at 1.25X (panel 1 and 3 from top, bar = 800 μM) or 20X (panel 2 and 4) magnification. (**D**) Quantification of Ki67 negative areas normalized to whole tumor areas by DAPI from three MYCMI-6- and three vehicle-treated mice. (**E**) microvascular density visualized by CD31 staining in the red channel at 1.25X magnification as in (**C**). (**F**) Quantification of CD31 staining normalized to whole tumor areas from three MYCMI-6- and three vehicle-treated mice. (**G**) Detection of MYCN:MAX protein interaction by isPLA performed on tumor tissue from mice treated with MYCMI-6 (upper panel) or vehicle (middle panel) using antibodies against MYCN and MAX. Representative images were taken at 40X magnification. (**H**) Quantification of MYCN:MAX isPLA signals in tumor tissue from MYCMI-6- and vehicle-treated mice, presented as average number of dots from four randomly chosen microscopic fields from MYCMI-6 treated mice normalized to corresponding values from vehicle-treated mice. SEM and *p*-values are indicated.

To analyze the effects of MYCMI-6 on tumor physiopathology *in vivo*, we utilized a mouse xenograft tumor model based on human *MYCN*-amplified SK-N-DZ neuroblastoma cells. Tumor cells were injected into the flank of athymic nude mice and allowed to form tumors after which MYCMI-6 or vehicle were administered by daily intraperitoneal injection at a dose of 20 mg/kg body weight for 1–2 weeks. TUNEL-staining of tumor sections revealed a dramatic increase in the extension of apoptotic areas in the tumors (Fig. 7A and B) and a significant increase in non-proliferative areas as determined by Ki67 staining (Fig. 7C and D) in tumors from MYCMI-6-treated mice compared with vehicle-treated mice. CD31 staining of endothelial cells revealed a significantly reduced microvascular density (MVD) in MYCMI-6-treated mice compared with vehicle-treated mice (Fig. 7C,E and F). Further, tumors from MYCMI-6-treated mice displayed signs of necrosis and hemorrhage and exhibited scar tissue to a larger extent than vehicle-treated mice (Suppl. Fig. S8A). Importantly, isPLA analysis showed a significant reduction in MYCN:MAX interaction in tumors from MYCMI-6- compared to vehicle-treated mice (Fig. 7G and H), indicating that MYCMI-6 reaches, and is active against, its target *in vivo*. MYCMI-6 administration was well tolerated in mice with only slight and temporal effects on body weight (Suppl. Fig. S8B).

In summary, administration of MYCMI-6 to MYCN-dependent tumor-bearing mice resulted in the reduction of MYCN:MAX interaction, tumor cell proliferation and MVD and in induction of apoptosis in the tumor tissue without causing severe side effects.

## Discussion

Targeting the interaction between MYC and its obligatory heterodimerization partner MAX, which is required for specific DNA-binding of the MYC:MAX complex, has been a desired goal ever since the discovery of MAX^1^. A number of efforts from several laboratories has been made to identify small molecule inhibitors targeting this interaction^15,16,26,34^, but many of these suffer from either poor efficacy, weak affinity for MYC, poor selectivity (or the lack of information about selectivity) and/or discrepancy between activity *in vitro* and *in vivo*. While these compounds were all identified through biochemical *in vitro* protein interaction assays or on the yeast2hybrid system^22,24,25,27,28,30^, we utilized a cell-based BiFC assay^35–37^ to screen for MYC:MAX interaction inhibitors. BiFC has previously been used successfully to identify inhibitors of p65:p50 NFkB subunit interactions in cells^51^. Cell-based screens have the advantage that hit compounds exhibit cell compatible features and interact with targets in their physiological state, thereby increasing the probability of finding hit molecules with higher bioactivity already at the screening level.

Here we describe three “hits” from our BiFC screening approach using a diverse library of small molecules from DTP/NIH (http://dtp.cancer.gov), MYCMI-6, MYCMI-11 and MYCMI-14. We could not find any obvious structural resemblance between the identified compounds, nor between these and previously described MYC:MAX inhibitors. The three MYCMIs were validated using a number of cell based and biophysical *in vitro* validation assays with the aim to identify molecules fulfilling the following criteria: (1) inhibition of MYC:MAX interaction in cells and *in vitro* with high efficacy and selectivity; (2) direct binding to MYC with high affinity and selectivity; (3) inhibition of tumor cell growth with high efficacy in a MYC-dependent manner; (4) MYC:MAX inhibition occurring under maintained MYC expression, thereby enabling specific pharmacological studies of the role of MYC:MAX in MYC biology; and (5) MYC:MAX inhibitory activity in tumor tissue *in vivo*. To our knowledge, no previously reported MYC:MAX inhibitor has fulfilled all of these five criteria.

Regarding the first criterion, MYCMI-6, MYCMI-11 and MYCMI-14 significantly inhibited MYC:MAX and MYCN:MAX interactions in human cells as verified by GLuc and isPLA assays (Figs 2 and 3), showing IC_50_s for inhibition of endogenous interactions of 1.5, 6 and 6 μM, respectively. Importantly, at active concentrations, the three compounds did not significantly inhibit cellular interactions between MAX and the intracellular bHLHZip MYC competitor MXD1 (MAD1), or hetero- or homodimer interactions between the bZip transcription factors FOS:JUN, GCN4:GCN4 and FRA1:JUN, which form similar dimeric α-helical structures as interacting bHLHZip proteins^52^ (Figs 2 and 3 and S2). As comparison, the IC_50_s of MYC:MAX inhibition in cells for previously reported compounds range from 10–100 μM in different cellular MYC:MAX interaction assays^25,27,32,53–55^. Apart from the MYCMIs, only 10058-F4 has been shown to be selective for MYC:MAX vs. MXD:MAX (in Y2H assays in yeast cells)^24^, and for most inhibitors data on selectivity in general in cells have been poor or are lacking.

In the biophysical PPI validation assays based on MST and SPR, MYCMI-6 inhibited the interaction between the recombinant bHLHZip domains of MYC and MAX with a K_d_ of 4.3 and 3.8 μM, respectively (Fig. 3), which is well in line with the IC_50_ of 1.5 μM in the cellular isPLA assay. In contrast, MYCMI-11 and MYCMI-14 were less effective at inhibiting the MYC:MAX interaction in the MST assay as well as in the isPLA. Further, MYCMI-6 did not inhibit MAX:MAX homodimerization at active concentrations, thus showing selectivity for MYC:MAX. As comparison, the reference 10058-F4 inhibited the MYC:MAX interaction less well, with an IC_50_ of >25 μM in the SPR assay. KJ-Pyr-9 has been shown to inhibit recombinant MYC:MAX:DNA and MAX:MAX:DNA interaction with an IC_50_ of approximately 10 and 30 μM, respectively, in fluorescence polarization assays^25,27^. In our hands, KJ-Pyr-9 did, however, not affect the MYC:MAX interaction in the SPR assay at concentrations up to 10 μM. The reason for this discrepancy between the results obtained by these two methods is unclear. Using a GLuc screening assay *in vitro*, Choi *et al*.^25^ identified the molecule sAJM589, which inhibited MYC:MAX interaction with an IC_50_ of 1.8 μM while not affecting MAX:MAX and other interactions at 20 μM, which is comparable with our data for MYCMI-6. However, inhibition of MYC:MAX in cells by sAJM589 seemed to require higher concentrations (10–30 μM), which coincided with downregulation of MYC expression^25^.

Regarding the second criterion, direct binding of MYCMIs to the recombinant bHLHZip domain of MYC *in vitro*, MST and SPR analyses showed that MYCMI-6 binds to MYC with a K_D_ of 1.6 ± 0.5 μM (Figs 4 and S4 and S5), again in good agreement with both MYC:MAX *in vitro* MST, SPR and cellular isPLA data. MYCMI-6 did not bind to MAX *in vitro* in the MST or SPR assays. Further, binding of MYCMI-6 to the bHLHZip domain of MAD1, the p53 core domain, BSA and YFP were negligible by SPR (Figs 4 and S5), thus demonstrating selectivity for MYC bHLHZip domain. By comparison, 10074-G5, 10058-F4 and the 10058-F4 analogue #474 were reported to have affinities for MYC with a K_D_ around 32, 40 and 17 μM as monitored by SPR, respectively^54^. The selectivity of these compounds with respect to direct binding to reference proteins by SPR has, however, not been described. KJ-Pyr-9, was reported to bind MYC, but not MAX, with a K_d_ of 6.5 nM in a Backscattering Interferometry (BSI) assay^27^. We could not detect any binding of KJ-Pyr-9 to MYC up to 8 μM in our SPR assay (Suppl. Fig. S5B). The reason for this discrepancy is unclear at present.

Park and coworkers previously published work describing MYCMI-6 (BXI-61) as an inhibitor of BCL-X_L:_BAK interactions by binding BCL-X_L_ at a concentration of 14 nM based on an *in vitro* fluorescence polarization assay^56^. We did not observe any binding of MYCMI-6 to BCL-X_L_ in the MST assay up to 10 μM, but detected some binding in the SPR assay (Suppl. Figs S4A and S5F), indicating that MYCMI-6 has at least a 5-fold lower affinity for BCL-X_L_ than for MYC. In the light of our findings, MYC:MAX inhibition is likely to contribute to the reported inhibition of lung tumor cell growth by MYCMI-6 in a mouse xenograft model^56^. MYCMI-6 (NSC354961) was also reported as a hit in *in vitro* screens for inhibitors of hTERT, S-adenosylmethionine decarboxylase (AdoMetDC) and of HDM2, in all these cases with approximately 10-fold higher IC_50_s than what we found for inhibition of MYC:MAX *in vitro*^57–59^. Notably, the hTERT inhibitory activity was found to be artificial due to unspecific binding to DNA *in vitro* at these concentrations^58^. While the activity towards AdoMetDC was not validated in cells^57^, MYCMI-6/NSC354961 did cause increased expression of p53 in cells at concentrations from 5 μM^59^. However, in the light of our findings, inhibition of MYC:MAX by MYCMI-6 is likely to contribute to p53 activation since inhibition of MYC by the dominant negative OmoMYC protein, which interferes with the MYC:MAX complex, is known to activate the p53 pathway^60^. One should also point out that in none of these studies direct binding of MYCMI-6 to the proposed target was demonstrated.

Regarding the third criterion, MYC-dependent tumor cell inhibition, MYCMI-6, MYCMI-11 and MYCMI-14 inhibited growth of neuroblastoma cells with *MYCN*-amplification to a significantly larger extent than *MYCN*-non-amplified cell lines, which correlated with the much higher expression of MYCN in the *MYCN*-amplified cell lines than of MYC in the non-amplified cell lines as determined by western blot analysis using pan-MYC antibodies (Fig. 5). MYCMI-6 was again clearly the most potent and selective of the MYCMIs. MYCMI-6 has GI_50_s as low as 0.5 μM in neuroblastoma and Burkitt’s lymphoma cells with deregulated MYCN/MYC, which is much more potent in comparison to most of the previously reported MYC:MAX inhibitors, such as 10058-F4, 10058-F4 analogues and 10074-G5. MYCMI-6 GI_50_s are well in agreement with the isPLA IC_50_ data and the effect on MYC target gene expression (Fig. 2). The cell growth inhibitory activities of certain analogues to 10074-G5, KJ-Pyr-9 and sAJM589 have been reported to be in a similar range as MYCMI-6 for some tumor cell lines^25–27^, but it is not clear if these effects are primarily linked to MYC:MAX inhibition or reduced MYC expression (or other effects). The MYC-dependent effects of MYCMI-6 was further supported by the significant correlation between the levels of MYC mRNA/protein and the response to MYCMI-6 within the NCI-60 tumor cell line panel, and the differential response to MYCMI-6 between MYC knockout and wt/reconstituted Rat1 cells (Fig. 6 and Suppl. Fig. 7). The former result also underscores that measuring MYC protein, not only mRNA, levels in tumors is important for predicting response to MYC inhibitors. Importantly, MYCMI-6 was not cytotoxic to primary normal human fibroblasts at concentrations that killed almost 100% of MYC-dependent tumor cells, thus demonstrating a promising therapeutic window for MYCMI-6.

Regarding the 4^th^ criterion, effects on MYC expression, it is important to keep in mind that many MYC:MAX inhibitors, including 10058-F4, 10074-G5 and sAJM589 down-regulate MYC-family protein levels, while MYCMI-6, MYCMI-11, MYCM-14 (Figs 2 and S6) and #474 do not^25,54,61^. For the former category, it is therefore difficult to distinguish whether the effects on MYC-dependent tumor cells is due to down-regulation of MYC expression, which will affect all MYC functions, or inhibition of MYC:MAX interaction, which will only affect MYC functions controlled by the MYC:MAX complex.

Inhibition of tumor cell growth *in vivo* using mouse tumor models has been reported previously for the MYC:MAX inhibitors 10058-F4, KJ-Pyr-9, KSI-3716 and Mycro3^27,28,61,62^. In these studies it was not demonstrated whether the compounds inhibited MYC:MAX interaction *in vivo*, but Mycro3 was shown to reduce expression of MYC. Utilizing a *MYCN*-amplified neuroblastoma xenograft tumor model, we could show that MYCMI-6 treatment significantly reduced MYCN:MAX interaction in tumor tissue, suggesting that MYCMI-6 reached and was active against its target *in vivo* (Fig. 7). This correlated significantly with massive induction of apoptosis and reduction of tumor cell proliferation in large areas of the tumors, which is typically observed after blocking MYC function in MYC ON/OFF and in the dominant negative Omomyc mouse tumor models^63–66^, as well as by 10008-F4 and by Mycro3 in neuroblastoma^61^ and pancreatic^62^ tumor models, respectively. Other features of MYCMI-6 treatment were reduction in microvascularity and signs of increased hemorrhage and necrosis, which might reflect collapse of tumor vasculature previously observed in the Omomyc tumor model^65^. The effects of MYCMI-6 treatment therefore resemble the effects of MYC depletion by genetic means in mouse tumor models. Importantly, MYCMI-6 treatment was well tolerated by the mice and did not result in severe side effects. As mentioned above, MYCMI-6 has previously been shown to inhibit lung tumor growth in a mouse xenograft model^56^. Further validation of the efficacy of MYCMI-6 in MYC-dependent mouse tumor models is needed in the future.

In conclusion, we have identified three new MYC:MAX inhibitors in a cell-based protein interaction screen. Among these, MYCMI-6 fulfills all the criteria we set up for a useful MYC:MAX inhibitor: (1) Potent and selective inhibition of MYC:MAX interaction in cells and *in vitro*; (2) selective direct binding to MYC; (3) inhibition of tumor cell growth in a MYC-dependent manner, all at similar single-digit micromolar concentrations, while sparing normal cells; (4) In contrast to many other MYC:MAX inhibitors, MYCMI-6 did not affect MYC or MYCN expression, and thus could be used as a specific molecular tool to explore role of the MYC:MAX and MYCN:MAX interactions in normal and tumor cells; and (5) Exhibiting MYC:MAX inhibitory activity in tumor tissue *in vivo*. For future research, it will be important to determine the precise mechanism of action of MYCMI-6 to enable the development of more potent and selective molecules towards clinically relevant direct MYC inhibitors.

## Methods

All experiments were performed in accordance with the guidelines and regulations of the Karolinska Institutet.

### Cell lines

HEK293A, MCF7, MDA-MB231, SK-N-F1, SK-N-RA, SK-N-AS, COS-7, HeLa, BJ cells, and Rat1 fibroblasts TGR-1 (wt), HO15.19 (MYC knockout) and HOmyc3 (MYC reconstituted HO15.19) were maintained in DMEM. The neuroblastoma cell lines Kelly, IMR-32 and SK-N-DZ, and the Burkitt’s lymphoma cell lines Mutu, Daudi and ST486 were kept in RPMI. SK-N-BE(2) cells were maintained in a 1:1 mixture of EMEM and F12 media, supplemented with 1% non-essential amino acid solution (NEAA) and 0.5% Glutamine. U2OS-MYC-ER cell lines were cultured in phenol-red free DMEM and treated with 100 nM 4-hydroxytamoxifen (HOT) (Sigma-Aldrich) to activate MYC-ER. IMR-90 was maintained in MEM. All media were supplemented with 10% FBS and 1% penicillin and streptomycin and kept at 37 °C and 5% CO_2_. All cell lines were mycoplasma free. Cell proliferation assays were performed as described in Supplementary Information.

### Compounds

A screening library of small molecules, 10 mM of each compound in DMSO delivered in a 96 well plate format, from the NCI/DTP Open Chemical Repository Diversity set library (http://dtp.nci.nih.gov) was used. After hit selection, three independent compound batches were obtained from NCI/DTP to confirm activity. In addition, compound MYCMI-6 was synthesized in larger quantities by Latvian Institute of Organic Synthesis, Riga, Latvia. All compounds were dissolved in DMSO (Sigma-Aldrich) to a final concentration of 10 mM, verified by mass spectrometry (LC-MS) and stored in −80 ^o^C for further use. DMSO, JQ1, 10058-F4 and 10074-G5 were purchased from Sigma-Aldrich. KJ-Pyr-9 was purchased from Cayman Chemical and was verified by LC-MS.

### Bimolecular fluorescence complementation assay (BiFC)

The BiFC assay using MYC fused to the C-terminal fragment of YFP and MAX fused to the N-terminal fragment of YFP has been described previously^38^. Fluorescence intensity was analyzed using an automated Axiovert 200M inverted microscope (Zeiss). Images were captured and processed using a Hamamatsu ORCA-ER camera together with software from Improvision (OpenLab 4.0.1). CMV-CFP was cotransfected as an internal standard and the ratio between YFP and CFP fluorescence intensity was calculated and normalized to the value of the DMSO treated cells.

### *Gaussia* luciferase protein fragment complementation assay (GLuc)

The protein fragment complementation assay using the *Gaussia* luciferase has been described^39^. 0.2–0.4 μg of each GLuc-construct (see Supplementary Information) together with 0.05–0.2 μg pCMV-Luc (Firefly luciferase) were transiently transfected into HEK293 or COS-7 cells in 6-well plates. 24 hours later cells were treated with compound (10–25 μM) or DMSO. After another 17 hours, the cells were harvested and lyzed in passive lysis buffer (Promega) supplemented with complete protease inhibitor (Roche). After 60 min incubation at room temperature 20 μM D-luciferin was added (substrate of Firefly luciferase) and luminescence was measured in a Lumat LB9501 (Berthold) or OmegaFluostar (BMG Labtech) luminometer. Directly after, *Gaussia* luciferase substrate Coelenterazine (Promega) was added to a final concentration of 20 μM in a mixture with “Stop n’ Glow” (Promega) and the luciferase activity was measured. The ratio between *Gaussia* and Firefly luciferase values were calculated and normalized to DMSO-treated control cells.

### isPLA

The isPLA assay has been described^40^. Antibodies used are listed in Supplementary Information. Briefly, cells were grown on collagen-coated chamber slides (Falcon), and treated with 1–10 μM of compounds, respectively, for 16 hours, then washed twice with PBS and fixed in ice cold methanol for 5–15 min at room temperature. Slides were washed in PBS with 0.05% Tween 20 and incubated in blocking buffer after which isPLA was performed using the Duolink® *in situ* PLA kit (Sigma-Aldrich) according to the manufacturer’s protocol. DNA was stained with DAPI. Incubation with primary antibodies were performed at +4 °C overnight. Images were taken using an Axiovert 200M inverted microscope (Zeiss) and fluorescent dots were quantified using semi-automated analysis in ImageJ (http://imagej.net) and averaged to number of dots per cell.

isPLA for tumor tissue was performed using 4% formaldehyde fixed paraffin embedded material. The tissue slides were deparaffinized at 65 °C for an hour, rehydrated in 1X Aqua De Par (Biocare Medical) and incubated at 80 °C for 10 min in a pressure chamber (Decloaking Chamber™ Biocare Medical). Slides were soaked in antigen retrieval buffer (10 mM sodium citrate, pH 6.0) and heated in the pressure chamber to 100 °C for 20 minutes, after which the temperature was lowered to 65 °C. Slides were removed from the chamber and cooled down to room temperature. The antigen retrieval buffer was replaced gradually by deionized H_2_O for 15 min. Slides were placed in blocking buffer (Duolink® isPLA kit) for 1.5 hours at 37 °C followed by an extra blocking step using the Mouse on Mouse (M.O.M.™) basic kit (Vector Laboratories) according to manufacturers’ protocol. isPLA was performed as above.

### Coimmunoprecipitation, western blot, immunohistochemistry and RT-qPCR analyses

These assays were performed essentially as described in^67,68^. For further information on immunohistochemistry, and on primers and antibodies, see Supplementary Information.

### Plasmids and recombinant proteins

For information about plasmids and production and purification of recombinant proteins see Supplementary Information.

### Microscale thermophoresis (MST)

MST was carried out on a Monolith NT.115 with blue/green filters, kindly provided by Luca Jovine, Karolinska Institutet Huddinge, according to manufacturer’s protocol (NanoTemper). MYCbHLHZip, MAXbHLHZip, BSA and BCL-X_L_ recombinant proteins and compounds were diluted and titrated in PBS supplemented with 0.05% Triton-X-114 or 0.05% Tween-20. Titration of molecules was carried out in 16 PCR tubes into which a fixed concentration of fluorescent molecule was added. The mixture was applied to capillaries (standard treated, NanoTemper), and scanned in the Monolith NT.115 to measure initial fluorescence of molecule. MST was induced and fluorescence was measured during 40 seconds. Double measurements were carried out (MST power of 20% and 40%, or 40% and 60%) for each sample. Delta fluorescence or relative fluorescence of fluorescent molecule normalized to control vehicle was plotted against titrated molecule concentration. The protein labeling kit GREEN-NHS (NanoTemper) was used to label primary amines of proteins according to manufacture’s protocol.

### Surface plasmon resonance (SPR)

The SPR experiments were performed at 25 °C using a Biacore T200 (GE Healthcare) instrument kindly provided by SciLifeLab Solna. An amino coupling procedure was used to immobilize protein on a CM5 sensor chip (GE Healthcare). Sensorgrams were generated by subtraction of the reference (blank immobilized) surface. All details will be described in Castell *et al*. (manuscript in preparation). In general, for the MYC:MAX interaction assay, 200–500 RU of MAXbHLHZip was immobilized and 100 nM MYCbHLHZip pre-incubated with compound in PBS supplemented with 0.05% Tween-20 and 1% DMSO was injected with a flow rate of 30 µl/min and allowed to dissociate. MAX was regenerated by injection of PBS buffer supplemented with Urea.

The MYC SPR assay was carried out as described above with MYCbHLHZip immobilized to a level of 800–1000 RU. For kinetic binding experiments, a Langmuir 1:1 binding event was applied using the Biacore T200 Evaluation Software 2.0 (GE Healthcare) to determine association (k_a_) and dissociation (k_d_) constants of the compound and to calculate affinity (K_D_) by the formula; K_D_ = k_d_/k_a_. Binding responses from equilibrium binding experiments were plotted against compound concentration and K_D_ values were determined at 50% of the theoretical Rmax with the formula Rmax = (MW analyte/MW ligand) × immobilized ligand level on the chip (RU) × stoichiometry (1:1). Same coupling method described above was used to immobilize the control proteins. MAX was immobilized to approximately 2000 RU, MAD to 600 RU, BSA to 1000 RU, p53 to 3000 RU and BCL-X_L_, to 500 RU.

### Tumor xenograft experiments

All animal protocols in this study were approved by the ethical committee for animal experiments of northern Stockholm (N241/15). Mice were maintained under pathogen-free conditions according to guidelines of the animal facility at MTC, Karolinska Institutet. 6–8 weeks old athymic nude mice (NMRI nude (NMRINU)) (female) (Taconic) were injected s.c. with 5 × 10^6^ *MYCN*-amplified SK-N-DZ neuroblastoma cells. When tumors reached a size of 100–200 mm^3^, mice started receiving treatment with MYCMI-6 or vehicle (10% Kolliphore, 5% Tween 80, 5% DMSO) administered daily via i.p. (200 μl). The last dose was administered 3 hrs before termination. Tumors were collected and frozen in OCT (Cryomount, Histolab) or fixed in buffered 4% formaldehyde solution (Histolab) and paraffin-embedded.

### Statistical analyses

Proportional data corresponding to GLuc, isPLA in cells for MYC:MAX and qPCR experiments in U2OS cell line were analyzed with a non-parametric Kruskal-Wallis test, while comparisons across treatments were performed with Bonferroni post hoc or Wilcoxon tests, to account for heteroscedasticity. Proportional data corresponding to MYCN:MAX isPLA and RT-qPCR experiments in neuroblastoma cell lines were initially transformed (square root and arcsine) and analyzed with generalized linear models (GLMs) with a normal distribution. Pairwise comparisons were performed with Bonferroni post hoc tests. Proportional data corresponding to isPLA *in vivo* experiments were log-transformed and analyzed with a GLM. The effect of MYC mRNA and protein levels on the response of the NCI-60 cancer cell lines to MYCMI-6 were analyzed with GLM assuming a normal distribution, while the hypothesis on the probability of a cell line with “high MYC” or “low MYC” protein levels to respond to MYCMI-6 was tested with the binomial exact test. The rest of the data were analyzed with the Student’s t-test. All analyses were carried out in R (v. 3.3.3; R Foundation for Statistical Computing, Vienna, AT), at a level of significance α = 0.05, with packages car, agricolae, multcomp and MASS.

## Electronic supplementary material

Supplemental Information

## References

[1] Blackwood EM, Eisenman RN (1991). Max: a helix-loop-helix zipper protein that forms a sequence-specific DNA-binding complex with Myc. Science.

[2] Dang CV (2012). MYC on the path to cancer. Cell.

[3] Eilers M, Eisenman RN (2008). Myc’s broad reach. Genes Dev.

[4] Larsson LG, Henriksson MA (2010). The Yin and Yang functions of the Myc oncoprotein in cancer development and as targets for therapy. Exp Cell Res.

[5] Meyer N, Penn LZ (2008). Reflecting on 25 years with MYC. Nat Rev Cancer.

[6] Nair, S. K. & Burley, S. K. X-ray structures of Myc-Max and Mad-Max recognizing DNA. Molecular bases of regulation by proto-oncogenic transcription factors. *Cell* **112**, 193–205, doi:S0092867402012849 [pii] (2003). 10.1016/s0092-8674(02)01284-9 12553908

[7] Kress TR, Sabo A, Amati B (2015). MYC: connecting selective transcriptional control to global RNA production. Nat Rev Cancer.

[8] Lin CY (2012). Transcriptional amplification in tumor cells with elevated c-Myc. Cell.

[9] Nie Z (2012). c-Myc is a universal amplifier of expressed genes in lymphocytes and embryonic stem cells. Cell.

[10] Sabo A (2014). Selective transcriptional regulation by Myc in cellular growth control and lymphomagenesis. Nature.

[11] Walz S (2014). Activation and repression by oncogenic MYC shape tumour-specific gene expression profiles. Nature.

[12] Gabay, M., Li, Y. & Felsher, D. W. MYC activation is a hallmark of cancer initiation and maintenance. *Cold Spring Harb Perspect Med* **4**, 10.1101/cshperspect.a014241 (2014). 10.1101/cshperspect.a014241 PMC4031954 24890832

[13] Sodir NM, Evan GI (2011). Finding cancer’s weakest link. Oncotarget.

[14] Castell A, Larsson LG (2015). Targeting MYC Translation in Colorectal Cancer. Cancer Discov.

[15] McKeown, M. R. & Bradner, J. E. Therapeutic strategies to inhibit MYC. *Cold Spring Harb Perspect Med* **4**, 10.1101/cshperspect.a014266 (2014). 10.1101/cshperspect.a014266 PMC4200208 25274755

[16] Whitfield JR, Beaulieu ME, Soucek L (2017). Strategies to Inhibit Myc and Their Clinical Applicability. Front Cell Dev Biol.

[17] Vassilev LT (2004). *In vivo* activation of the p53 pathway by small-molecule antagonists of MDM2. Science.

[18] Filippakopoulos P (2010). Selective inhibition of BET bromodomains. Nature.

[19] Tse C (2008). ABT-263: a potent and orally bioavailable Bcl-2 family inhibitor. Cancer Res.

[20] Arkin MR, Tang Y, Wells JA (2014). Small-molecule inhibitors of protein-protein interactions: progressing toward the reality. Chemistry & biology.

[21] Nero TL, Morton CJ, Holien JK, Wielens J, Parker MW (2014). Oncogenic protein interfaces: small molecules, big challenges. Nature reviews. Cancer.

[22] Berg T (2002). Small-molecule antagonists of Myc/Max dimerization inhibit Myc-induced transformation of chicken embryo fibroblasts. Proc Natl Acad Sci USA.

[23] Kiessling A, Sperl B, Hollis A, Eick D, Berg T (2006). Selective inhibition of c-Myc/Max dimerization and DNA binding by small molecules. Chem Biol.

[24] Yin X, Giap C, Lazo JS, Prochownik EV (2003). Low molecular weight inhibitors of Myc-Max interaction and function. Oncogene.

[25] Choi SH (2017). Targeted Disruption of Myc-Max Oncoprotein Complex by a Small Molecule. ACS Chem Biol.

[26] Fletcher S, Prochownik EV (2015). Small-molecule inhibitors of the Myc oncoprotein. Biochim Biophys Acta.

[27] Hart JR (2014). Inhibitor of MYC identified in a Krohnke pyridine library. Proc Natl Acad Sci USA.

[28] Jeong KC (2014). Intravesical instillation of c-MYC inhibitor KSI-3716 suppresses orthotopic bladder tumor growth. J Urol.

[29] Jung KY (2015). Perturbation of the c-Myc-Max protein-protein interaction via synthetic alpha-helix mimetics. J Med Chem.

[30] Kiessling A, Wiesinger R, Sperl B, Berg T (2007). Selective inhibition of c-Myc/Max dimerization by a pyrazolo[1,5-a]pyrimidine. ChemMedChem.

[31] Mo H, Henriksson M (2006). Identification of small molecules that induce apoptosis in a Myc-dependent manner and inhibit Myc-driven transformation. Proc Natl Acad Sci USA.

[32] Wang H (2007). Improved low molecular weight Myc-Max inhibitors. Mol Cancer Ther.

[33] Wang H (2015). Direct inhibition of c-Myc-Max heterodimers by celastrol and celastrol-inspired triterpenoids. Oncotarget.

[34] Prochownik EV, Vogt PK (2010). Therapeutic Targeting of Myc. Genes Cancer.

[35] Hu CD, Chinenov Y, Kerppola TK (2002). Visualization of interactions among bZIP and Rel family proteins in living cells using bimolecular fluorescence complementation. Mol Cell.

[36] Kerppola TK (2006). Complementary methods for studies of protein interactions in living cells. Nat Methods.

[37] von der Lehr, N. *et al*. The F-box protein Skp2 participates in c-Myc proteosomal degradation and acts as a cofactor for c-Myc-regulated transcription. *Mol Cell* 11, 1189–1200, doi:S109727650300193X [pii] (2003). 10.1016/s1097-2765(03)00193-x 12769844

[38] Grinberg AV, Hu CD, Kerppola TK (2004). Visualization of Myc/Max/Mad family dimers and the competition for dimerization in living cells. Mol Cell Biol.

[39] Remy I, Michnick SW (2006). A highly sensitive protein-protein interaction assay based on Gaussia luciferase. Nat Methods.

[40] Soderberg O (2006). Direct observation of individual endogenous protein complexes *in situ* by proximity ligation. Nat Methods.

[41] Ayer DE, Kretzner L, Eisenman RN (1993). Mad: a heterodimeric partner for Max that antagonizes Myc transcriptional activity. Cell.

[42] Larsson LG, Pettersson M, Oberg F, Nilsson K, Luscher B (1994). Expression of mad, mxi1, max and c-myc during induced differentiation of hematopoietic cells: opposite regulation of mad and c-myc. Oncogene.

[43] Adhikary S, Eilers M (2005). Transcriptional regulation and transformation by Myc proteins. Nat Rev Mol Cell Biol.

[44] Seidel SA (2013). Microscale thermophoresis quantifies biomolecular interactions under previously challenging conditions. Methods.

[45] Schasfoort, R. B. M. & Tudos, A. J. *Handbook of surface plasmon resonance* (RSC Pub., 2008).

[46] Zimmerman MW (2018). MYC Drives a Subset of High-Risk Pediatric Neuroblastomas and Is Activated through Mechanisms Including Enhancer Hijacking and Focal Enhancer Amplification. Cancer Discov.

[47] Jung M (2017). A Myc Activity Signature Predicts Poor Clinical Outcomes in Myc-Associated Cancers. Cancer Res.

[48] Bhat M (2015). Targeting the translation machinery in cancer. Nat Rev Drug Discov.

[49] Farrell, A. S. & Sears, R. C. MYC degradation. *Cold Spring Harb Perspect Med* **4**, 10.1101/cshperspect.a014365 (2014). 10.1101/cshperspect.a014365 PMC3935390 24591536

[50] Mateyak MK, Obaya AJ, Adachi S, Sedivy JM (1997). Phenotypes of c-Myc-deficient rat fibroblasts isolated by targeted homologous recombination. Cell Growth Differ.

[51] Yu H (2003). Measuring drug action in the cellular context using protein-fragment complementation assays. Assay Drug Dev Technol.

[52] Baxevanis AD, Vinson CR (1993). Interactions of coiled coils in transcription factors: where is the specificity?. Curr Opin Genet Dev.

[53] Clausen DM (2010). *In vitro* cytotoxicity and *in vivo* efficacy, pharmacokinetics, and metabolism of 10074-G5, a novel small-molecule inhibitor of c-Myc/Max dimerization. J Pharmacol Exp Ther.

[54] Muller I (2014). Targeting of the MYCN protein with small molecule c-MYC inhibitors. Plos One.

[55] Raffeiner P (2014). *In vivo* quantification and perturbation of Myc-Max interactions and the impact on oncogenic potential. Oncotarget.

[56] Park D (2013). Novel small-molecule inhibitors of Bcl-XL to treat lung cancer. Cancer Res.

[57] Brooks WH (2007). *In silico* chemical library screening and experimental validation of a novel 9-aminoacridine based lead-inhibitor of human S-adenosylmethionine decarboxylase. J Chem Inf Model.

[58] Sassano MF, Schlesinger AP, Jarstfer MB (2012). Identification of G-Quadruplex Inducers Usinga Simple, Inexpensiveand Rapid High Throughput Assay, and TheirInhibition of Human Telomerase. Open Med Chem J.

[59] Weissman, A. M. *et al*. Inhibiting Hdm2 and ubiquitin-activating enzyme: targeting the ubiquitin conjugating system in cancer. *Ernst Schering Found Symp Proc*, 171–190 (2008). 10.1007/2789_2008_108 19202599

[60] Soucek L (2013). Inhibition of Myc family proteins eradicates KRas-driven lung cancer in mice. Genes Dev.

[61] Zirath H (2013). MYC inhibition induces metabolic changes leading to accumulation of lipid droplets in tumor cells. Proc Natl Acad Sci USA.

[62] Stellas, D. *et al*. Therapeutic effects of an anti-Myc drug on mouse pancreatic cancer. *J Natl Cancer Inst* **106**, 10.1093/jnci/dju320 (2014). 10.1093/jnci/dju320 25306215

[63] Felsher, D. W. & Bishop, J. M. Reversible tumorigenesis by MYC in hematopoietic lineages. *Mol Cell* **4**, 199–207, doi:S1097-2765(00)80367-6 [pii] (1999). 10.1016/s1097-2765(00)80367-6 10488335

[64] Jain, M. *et al*. Sustained loss of a neoplastic phenotype by brief inactivation of MYC. *Science* **297**, 102–104, 10.1126/science.1071489 297/5578/102 [pii] (2002). 10.1126/science.1071489 12098700

[65] Sodir NM (2011). Endogenous Myc maintains the tumor microenvironment. Genes Dev.

[66] Soucek L (2008). Modelling Myc inhibition as a cancer therapy. Nature.

[67] Bahram F (2016). Interferon-gamma-induced p27KIP1 binds to and targets MYC for proteasome-mediated degradation. Oncotarget.

[68] Tabor V, Bocci M, Alikhani N, Kuiper R, Larsson LG (2014). MYC synergizes with activated BRAFV600E in mouse lung tumor development by suppressing senescence. Cancer Res.

