# Supplementary material for: A selective high affinity MYC-binding compound inhibits MYC:MAX interaction and MYC-dependent tumor cell proliferation
Source: Sci Rep. 2018 Jul 3;8:10064. doi: 10.1038/s41598-018-28107-4 (PMC6030159; doi:10.1038/s41598-018-28107-4)
Supplement: Supplementary file 1 — Supplemental Information [file 41598_2018_28107_MOESM1_ESM.pdf]

Supplemental information

A selective high affinity MYC-binding compound inhibits MYC:MAX interaction and MYC-dependent tumor cell proliferation

Alina Castell[1]*, Qinzi Yan[1]*, Karin Fawkner[1,2]*, Per Hydbring[1,3], Fan Zhang[1], Vasiliki Verschut[1], Marcela Franco[1], Siti Mariam Zakaria[1], Wesam Bazzar[1], Jacob Goodwin[1], Giovanna Zinzalla[1] and Lars-Gunnar Larsson[1,4]

Supplemental Methods

Plasmids. The BiFC plasmids MYC-YFP155-238 (MYC-YFP-C) MYCΔbHLHZip-YFP155-238 (MYCΔLZ-YFP-C) and MAX-YFP1-154 (MAX-YFP-N), FOS-YFP-N and JUN-YFP-C as well as full-length pCMV-CFP were kindly provided by T. Kerppola (Univ. of Michigan, Ann Arbor). Zip-hGLuc(1) (containing GCN4 and the N-terminal part of *Gaussia* luciferase) and Zip-hGLuc(2) (containing GCN4 and the C-terminal part of *Gaussia* luciferase) [1], kindly provided by S. Michnick (University of Montreal, Montreal), were used to create MYC-GLuc-C, MYCN-GLuc-C, MYCΔZip-GLuc-C, MAD-GLuc-C and MAX-GLuc-N by replacing the existing GCN4 gene with full-length MYC, MYC with deletion of the leucine zipper domain, full-length MYCN, full-length MXD1(MAD1) or full-length MAX cDNA, respectively. Full length Firefly luciferase (pCMV-Luc) was a kind gift from B. Lüscher, RWTH, Aachen. N-terminal 6xHis-tagged constructs MYCbHLHZip-mTurquoise and MAXbHLHZip-eYFP were cloned into pET28a, and both cloned as described in[2]. N-terminal 6xHis-tagged constructs MYCbHLHZip and MAXbHLHZip were cloned into pET28a.

Antibodies. Antibodies used for isPLA for cell cultures were C-33 α-MYC or B8.4.B α-MYCN combined with C-17 α-MAX or H-50 α-FRA1 (all Santa Cruz Biotechnology) combined with 2315S α-JUN (Cell Signaling Technology), or control DBD α-Gal4 antibody (Santa Cruz), all diluted 1:50. MYCN NCM II 100 (Santa Cruz) and MAX 101271 (Abcam)

antibodies at a 1:50 dilution were used for MYCN:MAX isPLA *in vivo*. Immunoprecipitation of MYC was performed with α-MYC N262 (Santa Cruz biotechnology) or α-MYC Y69 (Abcam) antibodies. The following antibodies were used for western blot: α-MYC N262 (Santa Cruz), α-MYC 9402S (Cell Signaling), α-MYC Y69 (Abcam), MYCN NCM II 100 (Santa Cruz), α-pan-MYC 195207 (Abcam), α-MAX 101271 (Abcam), α-Actin (A2228; Sigma-Aldrich or AC-15, Sigma). For staining of tumor tissue sections, the Ki67 Ab 16667 (Abcam), (dilution 1/500) and the CD31 Ab 553370 (BD Pharmingen), dilution 1/200, were used.

Primers for RT-qPCR. The following forward (FW) and reverse (REV) sequences of human primers were used:
GAPDH FW: ACATCGCTCAGACACCATG, REV: TGTAGTTGAGGTCAATGAAGGG, ODC1 FW: TCTGCTTGATATTGGCGGTG, REV: GGCTCAGCTATGATTCTCACTC, CR2 FW: GGGTTTTCTTGGCTCTCGTC, REV: CCTTATCACGGTACCAACAGC, RGS16 FW: CTGCGATACTGGGAGTACTGG, REV: CCACCCCAGCACATCTTC
CAD FW: CACTGAGCATGGCGTCAA, REV: AGCTGCTCCAGGATGCTC
GLUT1 FW: AGGACATCCAGGGTAGC, REV: GGTTGTGCCATACTCATGACC
HK2 FW: TCCCCTGCCACCAGACTA, REV: TGGACTTGAATCCCTTGGTC
LMO3 FW: GCTCCACCCTGTACACTAAAG, REV: ATCACCATCTCAAAGGCAGG
PFKM FW: GCCATCAGCCTTTGACAGA, REV: CTCCAAAAGTGCCATCACTG
TPI1 FW: GTTGGGGGAAACTGGAAGAT, REV: TAGGGGGAGCACAAACCA

Recombinant proteins. Recombinant proteins containing His-tagged N-termini were over-expressed in *E. coli BL21* (DE3) bacteria (Stratagene) at 37 °C in 2XTY, or LB, media with kanamycin. MAX proteins and MYC fluorescent fusion proteins were purified on a Ni-NTA (Qiagen) affinity bench column, or using a HisTrap HP column (5 mL) with an ÄKTA system. The purifications were carried out according to manufacturer instructions. Other MYC proteins were purified in denaturing conditions as described above. All the proteins were dialyzed against PBS, pH 7, at 5 °C overnight. The purity of the proteins was confirmed by SDS-PAGE analysis and Mass Spectrometry analysis. Lyophilized YFP and CFP were purchased from Medical and Biological Laboratories Co and Bovine serum albumin (BSA) was included in the MST kit from NanoTemper. BCL-X_L was purchased from Abcam, GST-MAD bHLHZip was purchased from Novus Biologicals. Proteins were dissolved in PBS, with or without 0.05%

Triton-X-114 (Sigma). Truncated p53 core protein was a kind gift from Klas Wiman, CCK, Karolinska Institutet.

Cell proliferation assays. Cell growth and viability was estimated in triplicates with WST-1 (Roche) or Resazurin sodium salt (Sigma-Aldrich) assays in medium at 37°C and 5% CO_2 for 2 hours after which absorbance or fluorescence, respectively, was measured with an Omega Fluostar (BMG Labtech) in a 96 well plate format. Cell counting was done in 96 well plates with CellTracker Green (ThermoFisher) for 30 min in the incubator, after which DAPI was added for 5 min. Images were taken in the green (FITC) and blue (DAPI) channels using a fluorescence microscope (ImageXpress Micro, Molecular Devices). For anchorage-independent growth assay, cells were suspended in 250 µl 0.35% SeaPlaque agarose (InVitro) and seeded into 24 well plates which had previously been coated with a bottom layer of 250 µl 0.7% agarose. After 16 days, the colonies were stained with 100 µg/ml MTT (Sigma) overnight and colonies were counted.

Immunohistochemistry. Apoptosis was visualized at the single-cell level on tumor cryosections using the TUNEL method using the *In Situ* Cell Death Detection Fluorescein-Roche kit and analyzed by fluorescence microscopy. Briefly, tumor cryosections were fixed in 4% paraformaldyde (PFA) and the assay was performed following the instructions from the manufacturer. Negative controls were run using the reagent without the TdT enzyme. Samples were mounted using Vectashield mounting medium (Vector) and DAPI was used as nuclear counterstaining. Cell proliferation and microvascular density (MVD) were evaluated through Ki67 and CD31staining, respectively, on tumor cryosections and detected by immunofluoresence. Visualization and image acquisition was done in a Zeiss microscope, and for panoramic views by a Vectra imaging system. For quantification, areas with positive TUNEL and Ki67 staining were measured using Image J software. For the analysis of MVD, the number of vessel structures per microscopic field was calculated.

Supplemental References

1 Remy, I. & Michnick, S. W. A highly sensitive protein-protein interaction assay based on Gaussia luciferase. *Nat Methods* **3**, 977-979, doi:10.1038/nmeth979 (2006).

2 Berg, T. *et al.* Small-molecule antagonists of Myc/Max dimerization inhibit Myc-induced transformation of chicken embryo fibroblasts. *Proc Natl Acad Sci U S A* **99**, 3830-3835, doi:10.1073/pnas.062036999 (2002).

3 Grinberg, A. V., Hu, C. D. & Kerppola, T. K. Visualization of Myc/Max/Mad family dimers and the competition for dimerization in living cells. *Mol Cell Biol* **24**, 4294-4308 (2004).

Supplementary Figure Legends

Supplemental Figure S1. Schematic presentations of MYC and MAX protein constructs. A) BiFC constructs MYC-YFP-N and MAX-YFP-C, containing full-length MYC and MAX fused with N-terminal and C-terminal fragments of yellow fluorescent protein (YFP), respectively [3]. In the MYC deletion mutant MYCΔbHLHZip-YFP-N, the bHLHZip domain, residues 352-432, has been truncated [3]. B) *Gaussia princeps* Luciferase (GLuc) constructs MYC-GLuc-C and MAX-GLuc-N, containing full-length MYC and MAX fused with N-terminal and C-terminal fragments of GLuc, respectively. MYC was truncated at residue 411-432 in order to produce the deletion mutant MYCΔZip-GLuc-C. C) His-tagged MYC and MAX bHLHZip domains were cloned into the bacterial expression vector pET28a for production of recombinant protein used in the MST and SPR assays.

Supplemental Figure S2. Effects of MYCMIs on FOS:JUN and MAD:MAX interactions in cells. A) BiFC assay of FOS:JUN interactions in cells. FOS-YFP-N and JUN-YFP-C were transiently expressed together with CMV-CFP in HEK293 cells. The cells were reseeded and treated with vehicle or 25 μM of each compound, after which fluorescence was measured 24 hours post-treatment and the YFP/CFP ratio calculated and normalized to DMSO-treated cells. B) MAD:MAX GLuc assay. MAD-GLuc-C and MAX-GLuc-N constructs were cotransfected into COS-7 cells together with CMV-Luc and treated with MYCMIs (12.5 μM) and analyzed as described in C).

Supplemental Figure S3. Effects of reference MYC:MAX inhibitory compounds in the MYC:MAX surface plasmon resonance (SPR) assay. SPR assay of MYC:MAX heterodimer formation. Reference surface subtracted sensorgrams are shown from one representative experiment. A) MYCbHLHZip was pre-incubated with 10058-F4 (as indicated) before injected onto the immobilized MAXbHLHZip protein. B) MYCbHLHZip was pre-incubated with KJ-Pyr-9 (as indicated) before injected onto the immobilized MAXbHLHZip protein.

Supplemental Figure S4. Characterization of the effect of MYCMI-6 on the MYC bHLHZip domain and control proteins by microscale thermophoresis (MST). A) MST of MYCMI-6 (3 μM) with recombinant BCL-X_L protein titrated as indicated. Four experiments are displayed.

B) MST of MYCMI-6 with bovine serum albumin (BSA) titrated as indicated. Three experiments are displayed. C) MST of labeled MYC (approximately 200 nM) titrated with MYCMI-6 as indicated. Data are shown as mean ± standard deviation of 5 biological repeats.

Supplemental Figure S5. Characterization of the binding of MYCMI-6 and other MYC:MAX inhibitory compounds on MYC and different control proteins by surface plasmon resonance (SPR). All sensorgrams are reference surface subtracted. A) SPR assay of MYCMI-6 injected over immobilized MYC in an equilibrium binding experiment where the theoretical Rmax is 23 RU. B) SPR assay of KJ-Pyr-9 injected over immobilized MYC. Theoretical Rmax is 29 RU. C) Sensorgram of MAX SPR assay. MYCMI-6 was injected at various concentrations over immobilized MAX bHLHZip (theoretical Rmax of 24 RU). D) Sensorgram of MAD SPR assay. MYCMI-6 was injected at various concentrations over immobilized GST-MAD bHLHZip (theoretical Rmax of 24 RU). E) Sensorgram of the BCL-X_L SPR assay. MYCMI-6 was injected at various concentrations over immobilized BCL-X_L (theoretical Rmax of 14 RU). F) Sensorgram of the BSA SPR assay. MYCMI-6 was injected at various concentrations over immobilized BSA (theoretical Rmax of 5 RU).

Supplemental Figure S6. Expression of MYCN and MAX proteins after MYCMI-6 treatment in MYCN-amplified neuroblastoma cells.
Western blot analysis of MYCN and MAX expression in SK-N-DZ and Kelly cells in response to MYCMI-6 (2.5 μM) or DMSO for 17 hours.

Supplemental Figure S7. Correlations between MYC levels in the NCI-60 human tumor cell line panel and the growth inhibitory response to MYCMI-6. A) MYC mRNA level data (x-axis) and GI50 response data to MYCMI-6 (y-axis) were extracted from the NIH database CellMiner™, see Supplemental Table S1, and plotted in a log graph. Red dots represent cell lines with elevated MYC protein levels. Orange dots represent cell lines with lower MYC protein levels. Black dots represent cell lines that have ABCB transporter mutations, which for this reason possibly have a reduced response to MYCMI-6. A general linear model was used to calculate correlation between MYC mRNA levels and response to MYCMI-6. P=0.1606761. B) Data was extracted as in A and supplemented with MYC protein data from the Novartis proteome scout SymAtlas Project and literature as described in Table 1. Cell lines were divided into higher or lower total levels of MYC (mRNA/protein) than average (x-axis) and their

response to MYCMI-6 (y-axis) and plotted in a bar graph. A general linear model was used to test for the relationship between MYC total levels and response to MYCMI-6 (P=0.0008924, X^2=11.0385).

Supplemental Figure S8. Histology of SK-N-DZ xenograft tumor tissue and body weight curves of mice under treatment with MYCMI-6 or vehicle. A) H&E staining of sections from formaldehyde-fixed paraffin-embedded SK-N-DZ tumor tissue obtained from mice treated with MYCMI-6 (left panels) or vehicle (right panels). Representative images are shown. Images were taken at 3X (upper panels) and 20X (lower panels) magnification. Black arrows: examples of necrotic tissue, black arrow heads: hemorrhage, white arrows: possible scar tissue and small white arrows: blood vessels. B) Body weight of mice under treatment with MYCMI-6 or vehicle taken every second day. Body weight was maintained.

Supplemental Figure S9. Uncropped, full length versions of blots presented in: A) main figure 1G, B) main Fig. 2E. "X" represents a compound that is not part of this work.

Supplemental Figure S10. Uncropped, full length versions of blots presented in: A) main Fig. 5A; B) Supplemental Fig. S6.

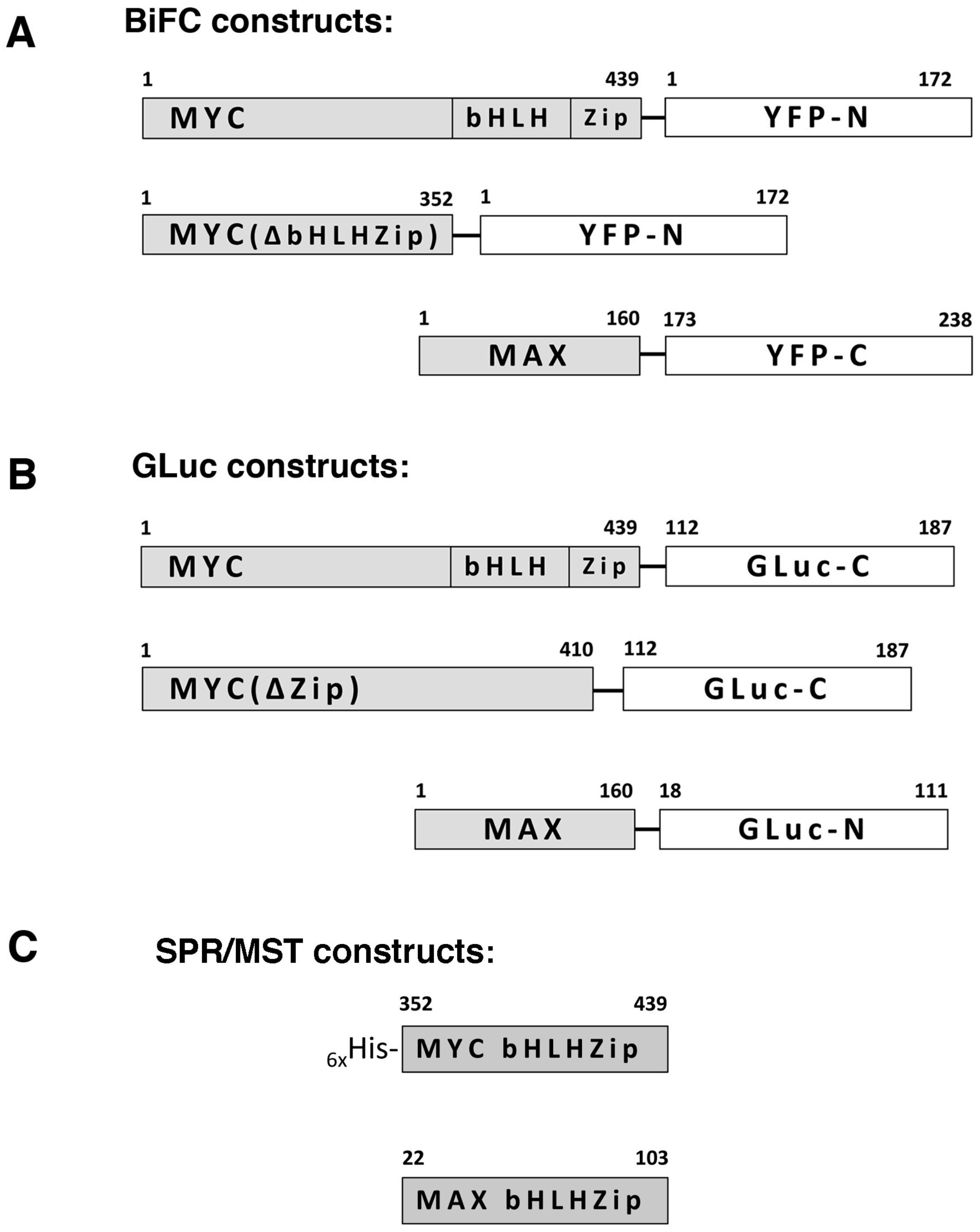
A
BiFC constructs:
1
439
1
172
MYC
bHLH
Zip
YFP-N
1
352
1
172
MYC(ΔbHLHZip)
YFP-N
1
160
173
238
MAX
YFP-C
B
GLuc constructs:
1
439
112
187
MYC
bHLH
Zip
GLuc-C
1
410
112
187
MYC(ΔZip)
GLuc-C
1
160
18
111
MAX
GLuc-N
C
SPR/MST constructs:
352
439
6xHis-
MYC bHLHZip
22
103
MAX bHLHZip

Suppl. Fig. S1

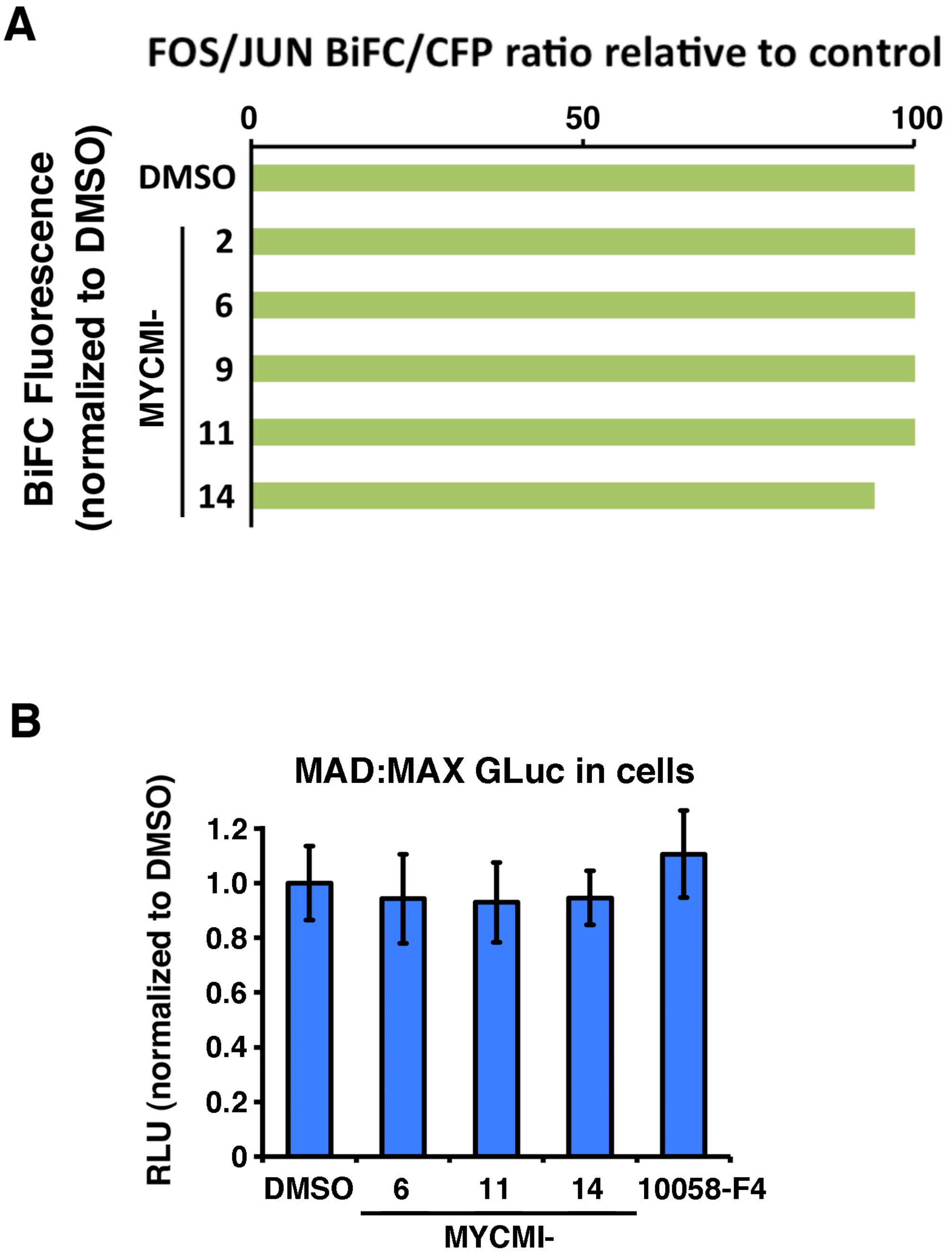
A
FOS/JUN BiFC/CFP ratio relative to control
0
50
100
BiFC Fluorescence (normalized to DMSO)
DMSO
MYCMI-
2
6
9
11
14
B
MAD:MAX GLuc in cells
RLU (normalized to DMSO)
1.2
1.0
0.8
0.6
0.4
0.2
0
DMSO
6
11
14
10058-F4
MYCMI-

Suppl. Fig. S2

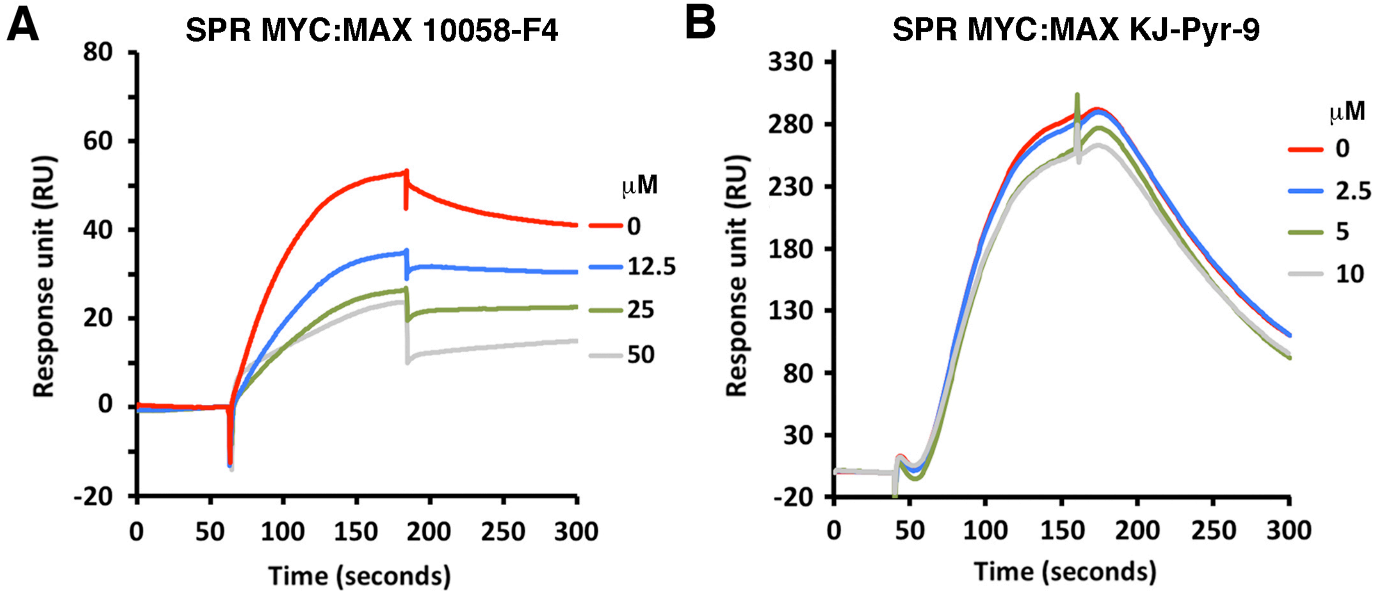
A
SPR MYC:MAX 10058-F4
80
60
40
20
0
-20
Response unit (RU)
0
50
100
150
200
250
300
Time (seconds)
μM
0
12.5
25
50
B
SPR MYC:MAX KJ-Pyr-9
330
280
230
180
130
80
30
-20
Response unit (RU)
0
50
100
150
200
250
300
Time (seconds)
μM
0
2.5
5
10

Suppl. Fig. S3

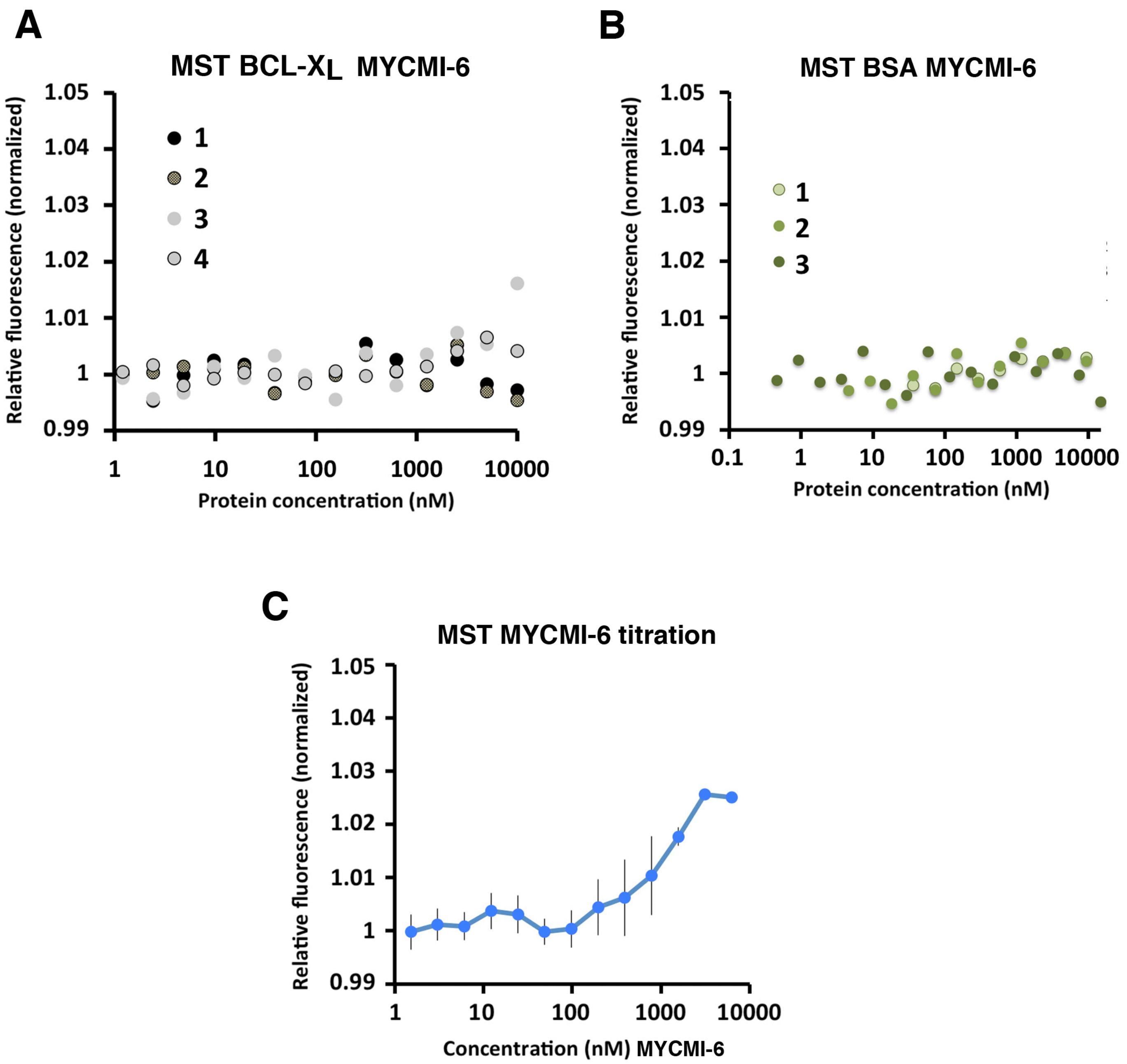
A
MST BCL-X_L MYCMI-6
Relative fluorescence (normalized)
1.05
1.04
1.03
1.02
1.01
1
0.99
1
2
3
4
1
10
100
1000
10000
Protein concentration (nM)
B
MST BSA MYCMI-6
Relative fluorescence (normalized)
1.05
1.04
1.03
1.02
1.01
1
0.99
1
2
3
0.1
1
10
100
1000
10000
Protein concentration (nM)
C
MST MYCMI-6 titration
Relative fluorescence (normalized)
1.05
1.04
1.03
1.02
1.01
1
0.99
1
10
100
1000
10000
Concentration (nM) MYCMI-6

Suppl. Fig. S4

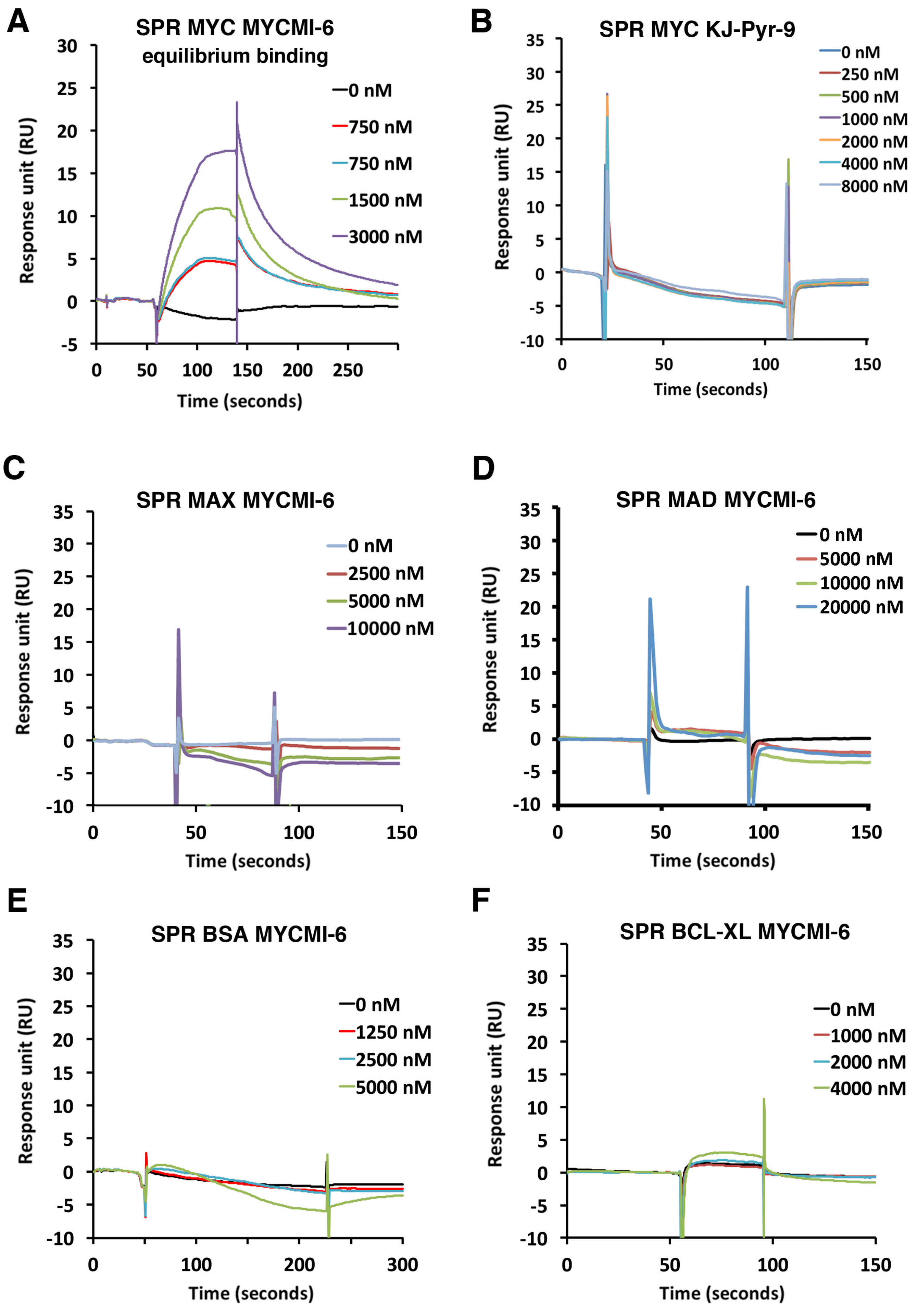
A
SPR MYC MYCMI-6
equilibrium binding
0 nM
750 nM
750 nM
1500 nM
3000 nM
Response unit (RU)
Time (seconds)
B
SPR MYC KJ-Pyr-9
0 nM
250 nM
500 nM
1000 nM
2000 nM
4000 nM
8000 nM
Response unit (RU)
Time (seconds)
C
SPR MAX MYCMI-6
0 nM
2500 nM
5000 nM
10000 nM
Response unit (RU)
Time (seconds)
D
SPR MAD MYCMI-6
0 nM
5000 nM
10000 nM
20000 nM
Response unit (RU)
Time (seconds)
E
SPR BSA MYCMI-6
0 nM
1250 nM
2500 nM
5000 nM
Response unit (RU)
Time (seconds)
F
SPR BCL-XL MYCMI-6
0 nM
1000 nM
2000 nM
4000 nM
Response unit (RU)
Time (seconds)

Suppl. Fig. S5

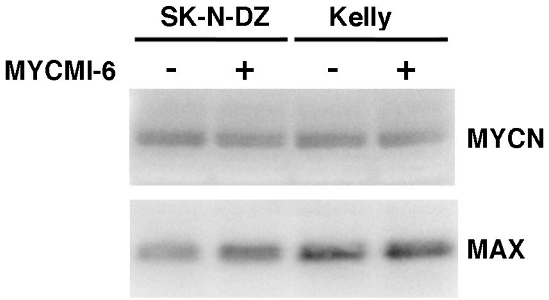

Suppl. Fig. S6

A

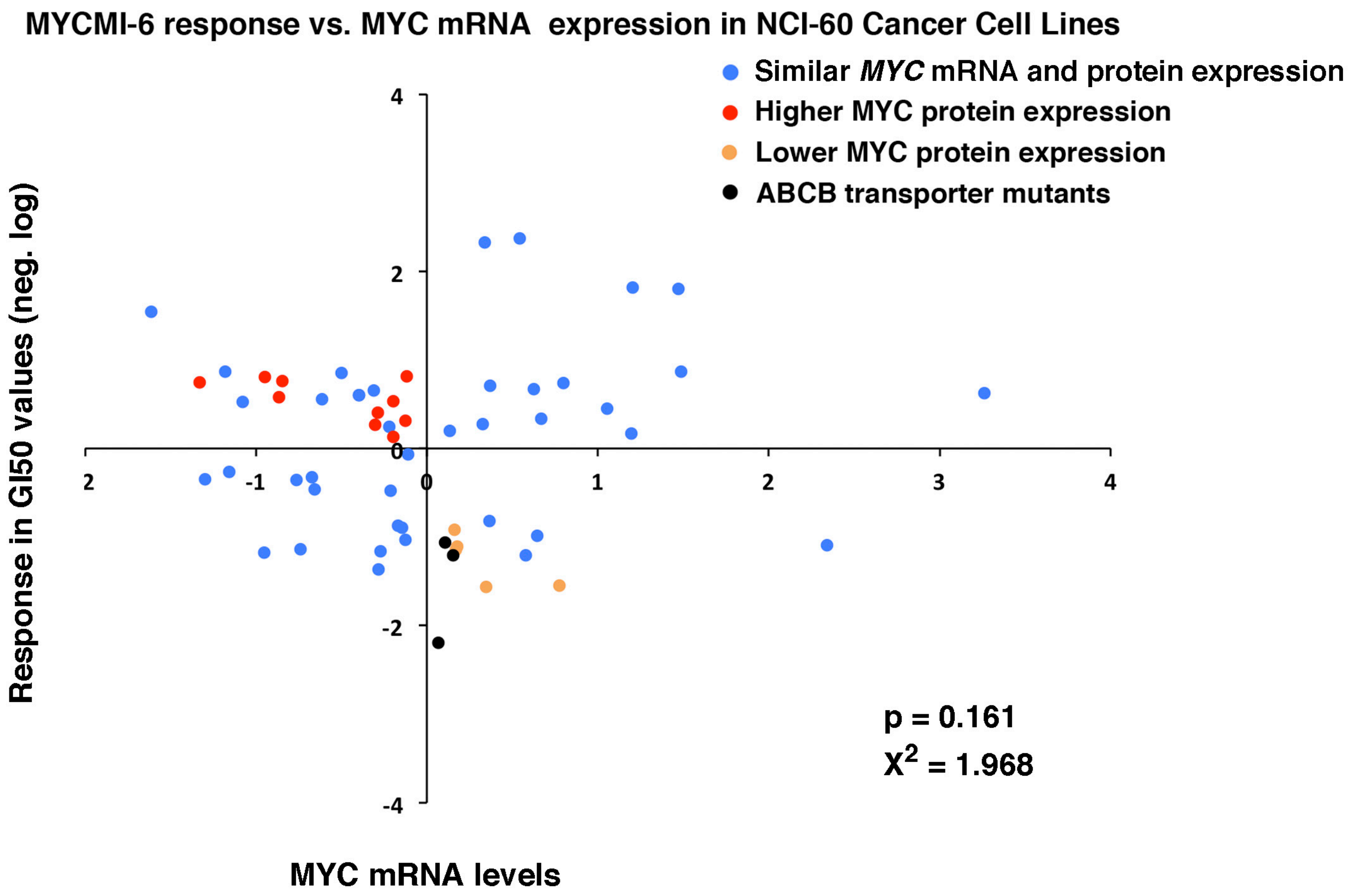

B

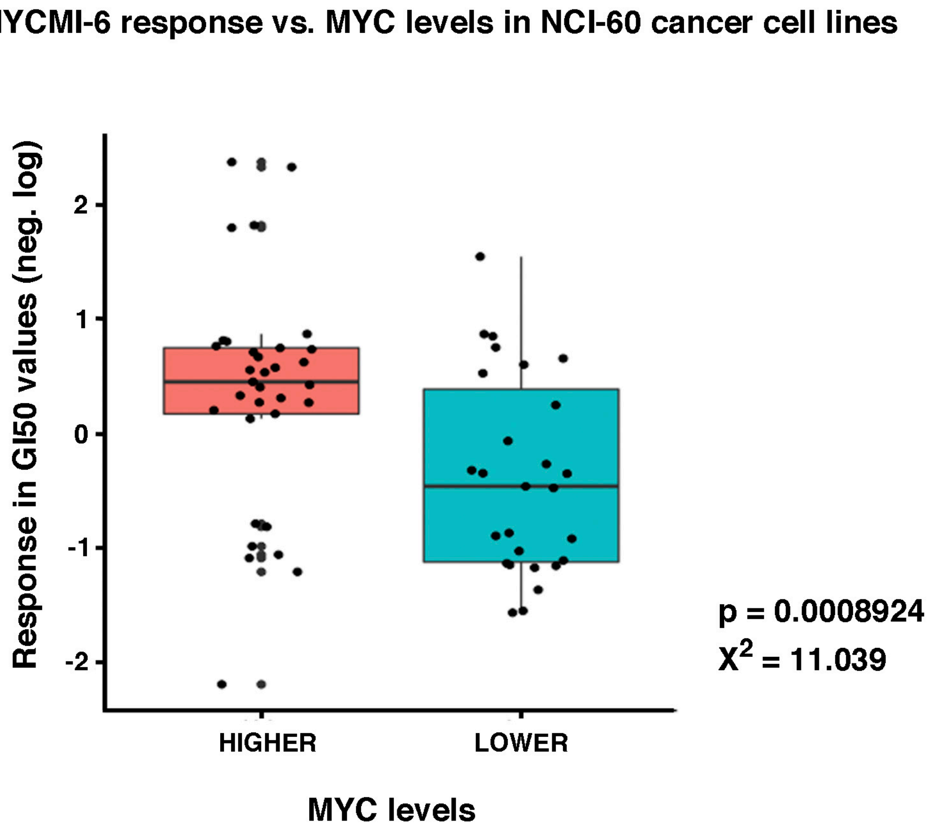

Suppl. Fig. S7

A

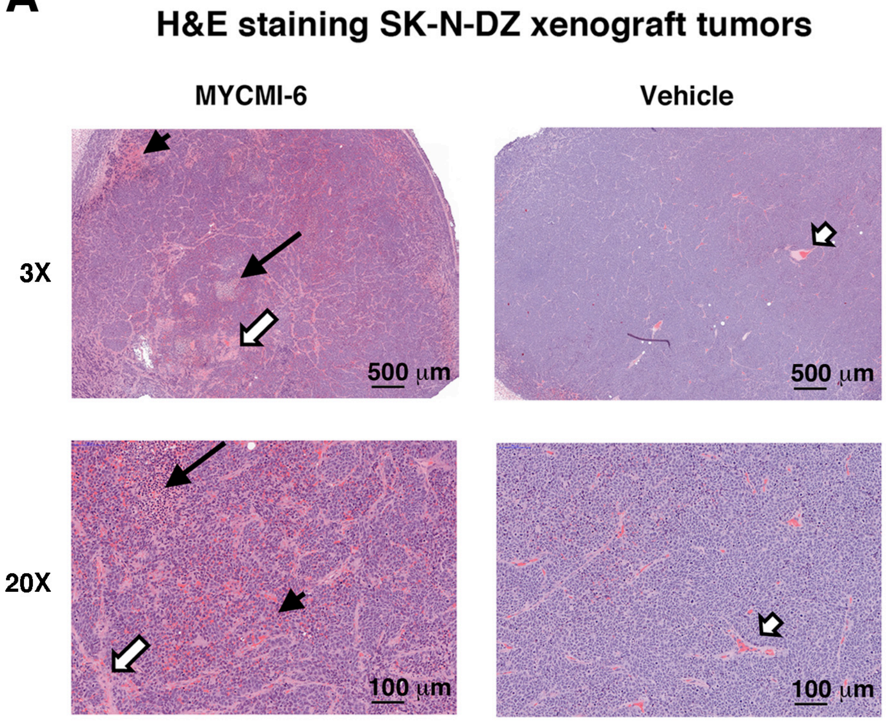

B

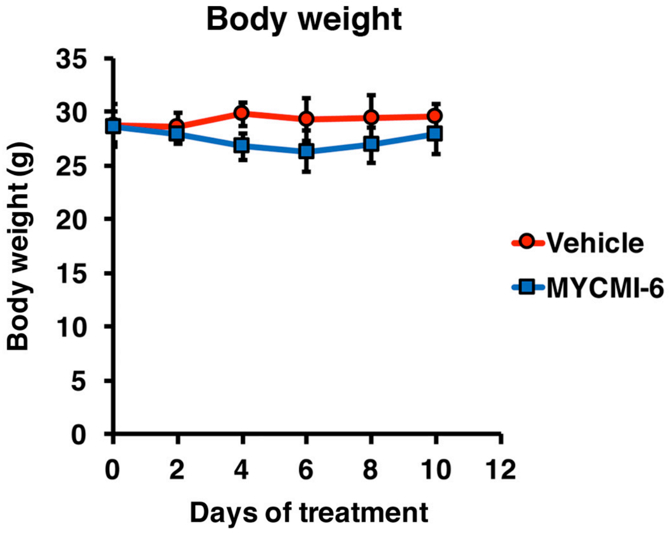

Suppl. Fig. S8

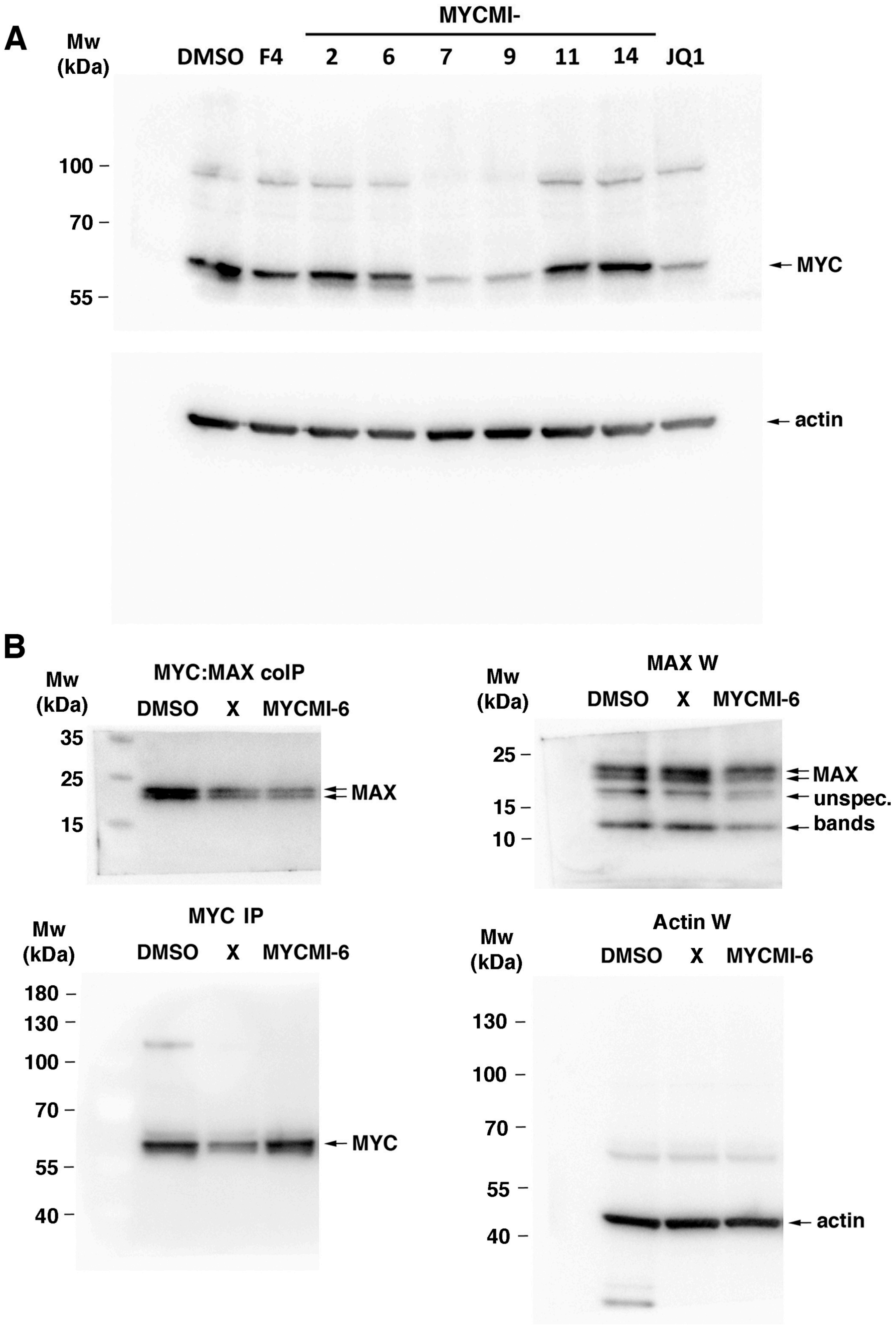
A
Mw (kDa)
MYCMI-
DMSO
F4
2
6
7
9
11
14
JQ1
100
70
55
MYC
actin
B
MYC:MAX coIP
Mw (kDa)
DMSO
X
MYCMI-6
35
25
15
MAX
MAX W
Mw (kDa)
DMSO
X
MYCMI-6
25
15
10
MAX
unspec.
bands
MYC IP
Mw (kDa)
DMSO
X
MYCMI-6
180
130
100
70
55
40
MYC
Actin W
Mw (kDa)
DMSO
X
MYCMI-6
130
100
70
55
40
actin

Suppl. Fig. S9

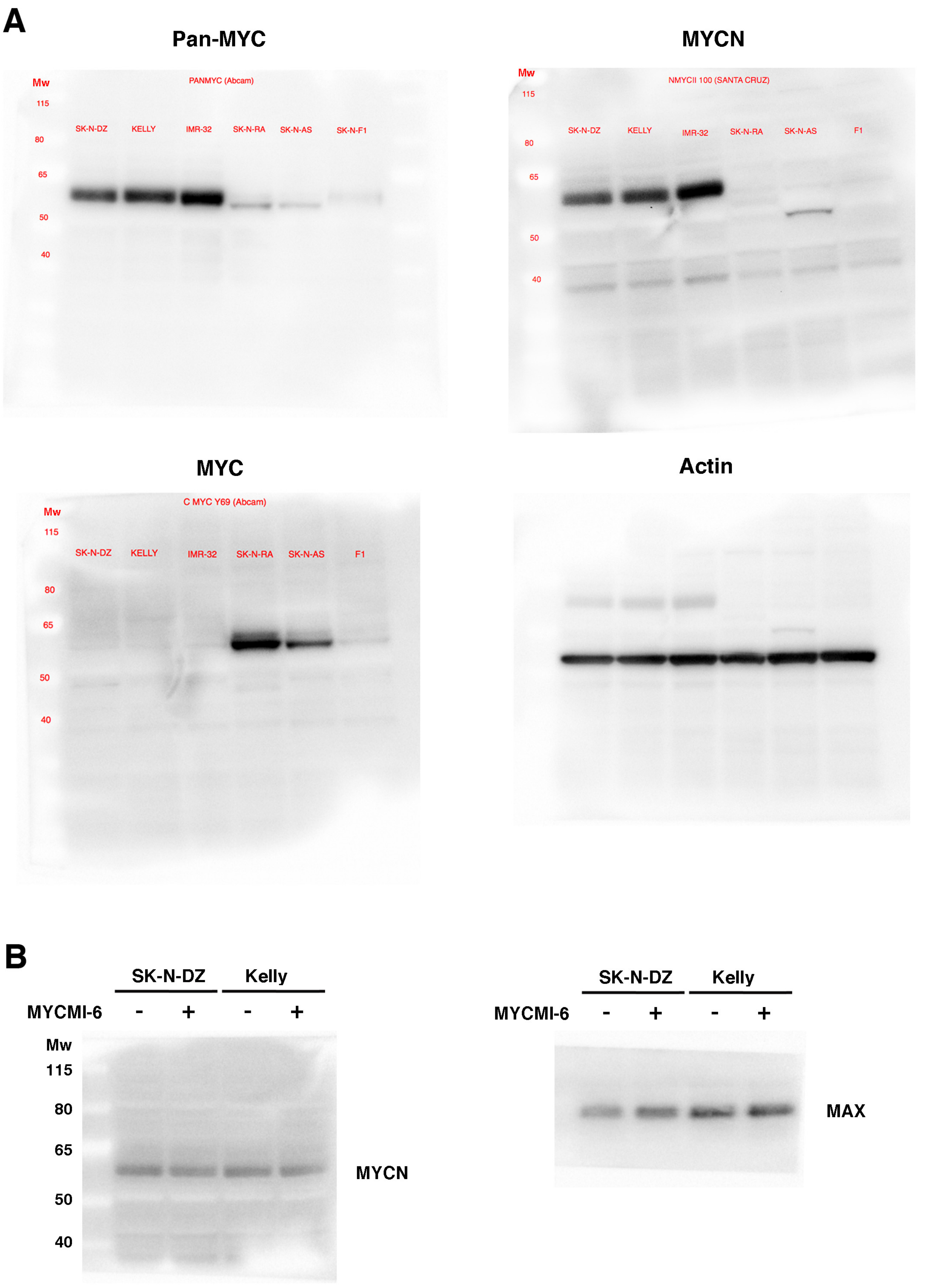

Suppl. Fig. S10

Supplemental Table S1. NCI-60 Cancer Cell Line data for MYCMI-6 drug activity vs. MYC mRNA and protein expression

		Cancer Cell lines	MYCMI-6 GI50 values[a]	MYC mRNA levels[b]	MYC protein levels[c]	ABCB mutants[d]	References[e]
Cancer cell lines with higher MYC levels	Responsive	**LE:CCRF-CEM**	2,373	0,542			
		**LE:SR**	2,328	0,338			
		**LE:K-562**	1,819	1,206			
		**ME:LOX IMVI**	1,798	1,471			
		**LE:MOLT-4**	0,87	1,486			
		**BR:MDA-MB-231**	0,813	-0,118	HIGHER		(1)
		**OV:OVCAR-3**	0,804	-0,946	HIGHER		(2)
		**CNS:SF-268**	0,764	-0,845	HIGHER		(3) NPS
		**BR:T-47D**	0,748	-1,328	HIGHER		(1, 4)
		**CO:HCT-116**	0,735	0,8			
		**CO:HCC-2998**	0,711	0,372			
		**CO:COLO 205**	0,669	0,626			
		**LE:HL-60(TB)**	0,622	3,261			
		**ME:SK-MEL-2**	0,576	-0,865	HIGHER		(5)
		**RE:SN12C**	0,554	-0,612	HIGHER		NPS
		**RE:RXF 393**	0,534	-0,197	HIGHER		NPS
		**CO:SW-620**	0,451	1,053			
		**LC:NCI-H522**	0,425	0,58			
		**OV:OVCAR-8**	0,404	-0,287	HIGHER		(6)
		**CO:KM12**	0,332	0,668			
		**ME:MDA-MB-435**	0,31	-0,125	HIGHER		(7)
		**BR:HS 578T**	0,269	-0,304	HIGHER		
		**BR:MCF7**	0,272	0,326			
		**CO:HT29**	0,202	0,136			
		**PR:PC-3**	0,171	1,195			
		**LC:NCI-H23**	0,129	-0,195	HIGHER		(8)
	Less responsive	**BR:BT-549**	-0,79	0,447			
		**RE:TK-10**	-0,816	0,368			
		**ME:SK-MEL-5**	-0,987	0,646			
		**ME:UACC-257**	-1,06	0,105		yes	COSMIC
		**LE:RPMI-8226**	-1,09	2,341			
		**OV:IGROV1**	-1,21	0,152		yes	COSMIC
		**OV:NCI/ADR-RES**	-2,194	0,067		yes	COSMIC
Cancer cell lines with lower MYC levels	Responsive	**LC:HOP-62**	0,248	-0,22			
		**LC:NCI-H322M**	0,525	-1,076			
		**CNS:SF-539**	0,601	-0,397			
		**ME:MDA-N**	0,656	-0,312			
		**CNS:SNB-19**	0,753	-1,366			
		**RE:786-0**	0,849	-0,498			
		**CNS:SNB-75**	0,868	-1,179			
		**CNS:U251**	1,546	-1,614			
	Less responsive	**CO:HCT-15**	-1,567	0,345	LOWER		(9), COSMIC
		**RE:CAKI-1**	-1,551	0,776	LOWER		NPS
		**RE:UO-31**	-1,368	-0,281			
		**CNS:SF-295**	-1,174	-0,952			
		**LC:NCI-H226**	-1,158	-0,273			

**LC:NCI-H460**	-1,149	0,17	LOWER	NPS
**OV:OVCAR-5**	-1,137	-0,74		
**ME:M14**	-1,111	0,178	LOWER	NPS
**RE:ACHN**	-1,028	-0,126		
**ME:SK-MEL-28**	-0,921	0,162	LOWER	NPS
**LC:A549/ATCC**	-0,895	-0,145		
**ME:UACC-62**	-0,87	-0,17		
**LC:HOP-92**	-0,477	-0,213		
**RE:A498**	-0,462	-0,657		
**OV:SK-OV-3**	-0,352	-0,763		
**LC:EKVX**	-0,348	-1,296		
**OV:OVCAR-4**	-0,322	-0,671		
**PR:DU-145**	-0,268	-1,155		
**ME:MALME-3M**	-0,066	-0,108		

a GI50 values of MYCMI-6 extracted from CellMiner (https://discover.nci.nih.gov/cellminer)
b MYC mRNA levels extracted from CellMiner (https://discover.nci.nih.gov/cellminer)
c MYC protein levels extracted from Novartis Proteome Scout (NPS) or other publicly available data, see references.
d Cell lines with ABCB transporter protein mutations from the COSMIC data base (http://cancer.sanger.ac.uk/cosmic)
e References:

1. Horiuchi D, *et al.* (2012) MYC pathway activation in triple-negative breast cancer is synthetic lethal with CDK inhibition. *J Exp Med* 209(4):679-696.
2. Seviour EG, *et al.* (2016) Functional proteomics identifies miRNAs to target a p27/Myc/phospho-Rb signature in breast and ovarian cancer. *Oncogene* 35(6):691-701.
3. Feng XD, *et al.* (2007) Analysis of pathway activity in primary tumors and NCI60 cell lines using gene expression profiling data. *Genomics Proteomics Bioinformatics* 5(1):15-24.
4. Kang J, Sergio CM, Sutherland RL, & Musgrove EA (2014) Targeting cyclin-dependent kinase 1 (CDK1) but not CDK4/6 or CDK2 is selectively lethal to MYC-dependent human breast cancer cells. *BMC Cancer* 14:32.
5. Zhuang D, *et al.* (2008) C-MYC overexpression is required for continuous suppression of oncogene-induced senescence in melanoma cells. *Oncogene* 27(52):6623-6634.
6. Ma Y, *et al.* (2015) Sequential treatment with aurora B inhibitors enhances cisplatin-mediated apoptosis via c-Myc. *J Mol Med (Berl)* 93(4):427-438.
7. Melkoumian ZK, Martirosyan AR, & Strobl JS (2002) Myc protein is differentially sensitive to quinidine in tumor versus immortalized breast epithelial cell lines. *Int J Cancer* 102(1):60-69.
8. Jo MJ, *et al.* (2015) Regulation of cancer cell death by a novel compound, C604, in a c-Myc-overexpressing cellular environment. *Eur J Pharmacol* 769:257-265.
9. Abe M, *et al.* (2011) Mechanisms of confluence-dependent expression of CD26 in colon cancer cell lines. *BMC Cancer* 11:51.
